# Black Phosphorus Degradation during Intercalation and Alloying in Batteries

**DOI:** 10.1021/acsnano.2c08776

**Published:** 2023-03-27

**Authors:** Samia Said, Zhenyu Zhang, Rebecca R. C. Shutt, Hector J. Lancaster, Dan J. L. Brett, Christopher A. Howard, Thomas S. Miller

**Affiliations:** † Electrochemical Innovation Lab, Department of Chemical Engineering, University College London, Torrington Place, London, WC1E 7JE, U.K.; ‡ The Faraday Institution, Quad One, Becquerel Avenue, Harwell Campus, Didcot, OX11 ORA, U.K.; § Department of Physics & Astronomy, University College London, Gower Street, London, WC1E 6BT, U.K.

**Keywords:** 2D nanomaterials, sodium ion battery, lithium ion battery, electrochemical atomic force microscopy, EC-AFM

## Abstract

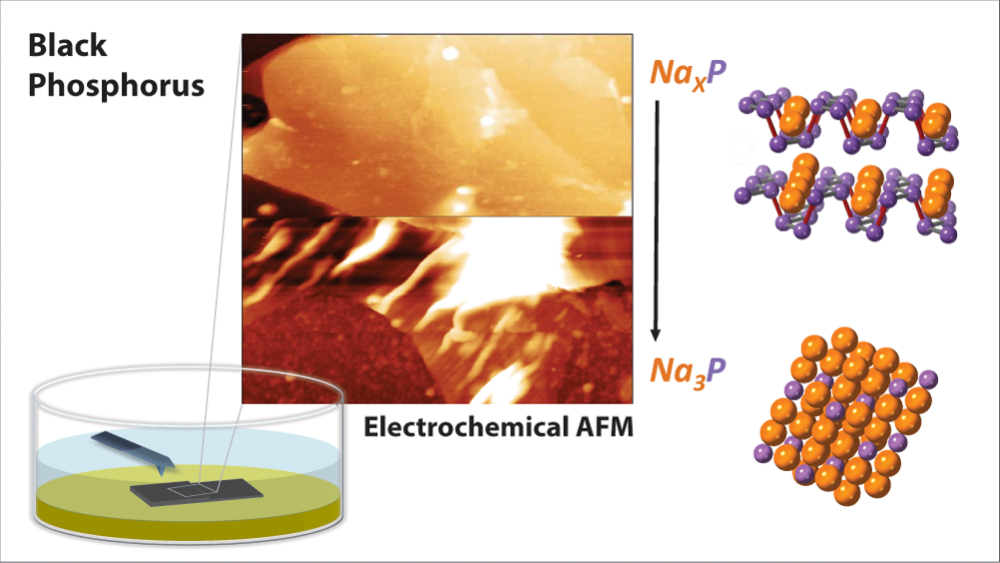

Numerous layered materials are being recognized as promising candidates for high-performance alkali-ion battery anodes, but black phosphorus (BP) has received particular attention. This is due to its high specific capacity, due to a mixed alkali-ion storage mechanism (intercalation-alloying), and fast alkali-ion transport within its layers. Unfortunately, BP based batteries are also commonly associated with serious irreversible losses and poor cycling stability. This is known to be linked to alloying, but there is little experimental evidence of the morphological, mechanical, or chemical changes that BP undergoes in operational cells and thus little understanding of the factors that must be mitigated to optimize performance. Here the degradation mechanisms of BP alkali-ion battery anodes are revealed through *operando* electrochemical atomic force microscopy (EC-AFM) and *ex situ* spectroscopy. Among other phenomena, BP is observed to wrinkle and deform during intercalation but suffers from complete structural breakdown upon alloying. The solid electrolyte interphase (SEI) is also found to be unstable, nucleating at defects before spreading across the basal planes but then disintegrating upon desodiation, even above alloying potentials. By directly linking these localized phenomena with the whole-cell performance, we can now engineer stabilizing protocols for next-generation high-capacity alkali-ion batteries.

Lithium-ion batteries (LIBs) are widely used energy storage devices; however, sodium-ion batteries (NIBs) are promising alternatives due to several advantages, including the high natural abundance of sodium, its lower cost and improved cell safety. While graphite is still the most widely utilized active material in LIB anodes, it offers poor performance in NIBs,^1^ which instead typically use hard carbon that has limited capacity (284 mAh g^–1^) and slow kinetics.^2^ Alternative anodes are being explored, including alloying materials such as Ge, Si, Sn, Pb, As, and Sb,^3−5^ which promise much higher volumetric and gravimetric energy densities; however, they suffer from a volume expansion during sodiation (e.g., 423% for the Sn–Na).^6^ This causes problems as it lowers Coulombic efficiency and complicates electrode engineering.

Many alternative van der Waals layered materials are being investigated as electrodes for alkali-ion batteries because of their very high storage capacities, excellent electrical conductivity, and low diffusion barriers.^7−10^ However, while ion storage proceeds via the same intercalation mechanism as graphite, this is often accompanied by alloying or conversion reactions that involve the breaking of bonds within the layers and associated irreversible losses. Intercalation-type materials include graphite and its derivatives,^11−19^ transition metal carbides (TMCs) i.e. M_*n*+1_X_*n*_ (where M = Sc, Ti, V, Cr, Zr, Nb, Mo, Hf, Ta; X = C, N; and typically *n* = 1, 2, or 3),^20−31^ and some transition metal oxides (TMOs) (where TM= Mo, Ti, Nb).^32−40^ However their available (theoretical) capacity, determined by the maximum number of vacant sites that can reversibly incorporate mobile ions, is limited by the preservation of the in-plane layered crystal structure of the host material. Typically this means the ratio of alkali-ions to active material must remain stoichiometrically far below 1, as higher levels of charging leads to undesirable chemical reactions. Examples of mixed alkali-ion storage materials include other transition metal oxides (TMO) (where TM= Co, Ni, Fe, Cu),^41,42^ transition metal dichalcogenides (TMD) e.g., MX_2_ (M = Mo, Sn, W; X = S, Se)^43−51^ and monoelemental structures such as black phosphorus (BP),^52−55^ that reach significantly higher stoichiometries, owing to their multielectron reactions which changes the bonding within the host.

BP is considered to be potentially promising for use in batteries, and experimental investigations of BP based anodes have demonstrated maximum capacities that approach the theoretical limits for BP in NIBs^54^ and LIBs.^56^ The electrochemical insertion of ions occurs in a two-step manner, first via intercalation, and then by an alloying reaction that delivers a high theoretical capacity (e.g., 2596 mAh g^–1^ for sodiation to Na_3_P).^55,53^ Additionally, of particular significance is the anisotropic behavior that arises from BP’s atomic structure, in which P–P bonds in each layer are of two different lengths. Bulk layered BP (structure shown in Figure 1) can therefore accommodate sodium ions within its interlayer spacing (3.08 Å vs 1.98 Å for graphite)^57−59^ and the “channels” formed by the armchair arrangement of the layers results in a low energy barrier (0.04 eV) for the diffusion of sodium along the [100] zigzag direction.^60^ Together this means BP can theoretically offer very high rate capabilities.

**Figure 1:**
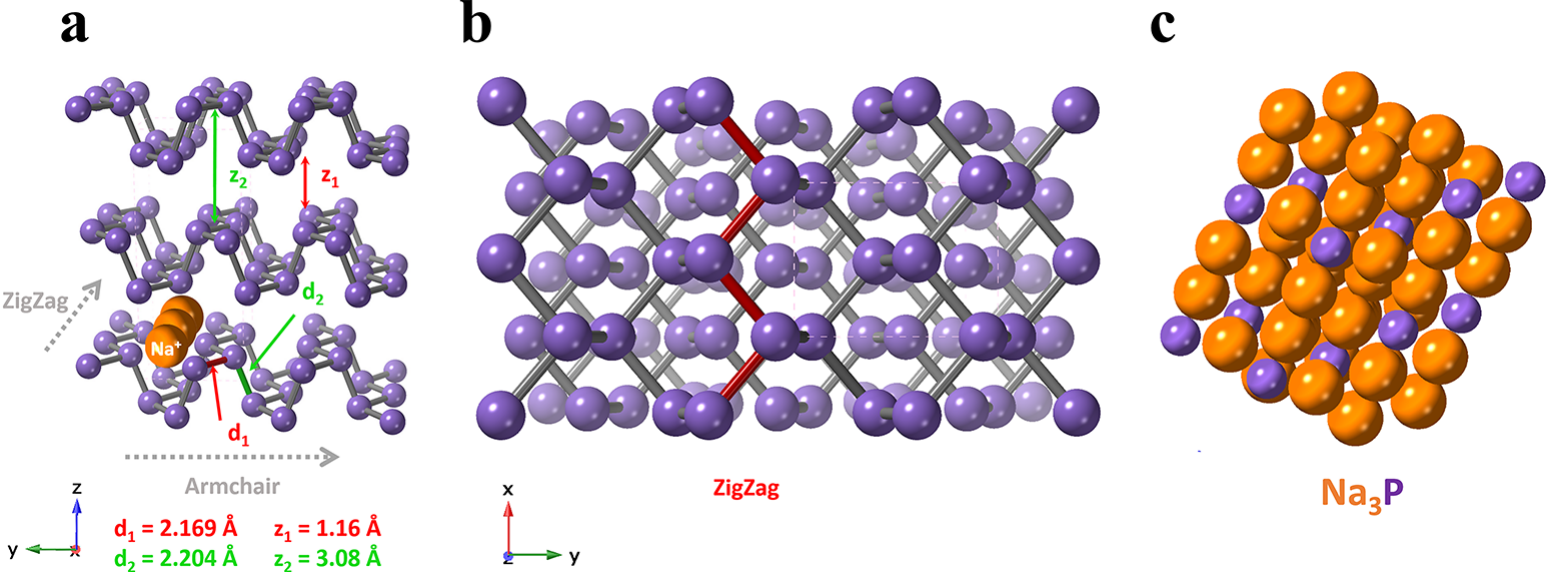
(a) Schematic of BP indicating sites of sodium-ion intercalation, showing the zigzag and armchair directions. (b) Top down view of a BP crystal showing the zigzag directions. (c) The alloy Na_3_P.

However, rate performance and cycling stability of BP-based batteries have been shown to be poor, in part due to its low intrinsic electrical conductivity.^61^ Nanosizing BP, either to form nanoparticles or exfoliated sheets, and hybridizing these with, or supporting on, conductive carbon has, however, shown some success in improving anode performance.^57,61−68^ It is claimed that the addition of graphene based structures, or similar, in hybrid materials adds strong mechanical backbones and electrical highways.^61,57^ Control of the solid electrolyte interphase (SEI) film formed on BP electrodes, through the use of electrolyte additives, has also been suggested as a route to achieve longer cycle life and higher reversibility.^69^ However, to date studies of BP in representative batteries provide little experimental evidence to directly link the overall electrochemical performance changes with structural or interphasial surface changes of the anode materials, at any scale.

In the case of sodiation, it is known that only a small proportion (∼150 mAh g^–1^) of the capacity of BP is due to intercalation, occurring between 1.5 and 0.6 V, to form Na_0.17_P (or NaP_6_), which is directly analogous to the LiC_6_ formed during the intercalation of graphite in LIBs. For graphite, if this stoichiometry is exceeded, metallic lithium can deposit, or plate, with potentially serious consequences for reliability and safety.^70^ Electrochemical sodiation of graphite, however, achieves much lower intercalation stoichiometries (≈NaC_186_), after which point Na deposition/plating tends to occurs.^71,72^ In contrast, a significant additional capacity can be achieved during sodiation of BP electrodes, due to a three-electron alloying redox reaction forming Na_3_P below ∼0.54 V. Similar to other mixed storage mechanism electrodes, such as Si and some TMOs, the alloying/conversion mechanisms are accompanied by a large volume expansion.^3,6,41,42^ For this family of materials, it is well-known that the extreme volumetric fluctuations can lead to reduced electrical contact (particle isolation) and particle-to-bulk level cracking, generating “dead” electrically insulated active material, although each will have its individual intricacies. Many of these processes act to stimulate continuous reformation of surface passivation layers which consumes Na (or Li in LIBs), and leads to an increase in the internal impedance, by reducing the electrochemical reactivity from through an accumulation of SEI products.^73^ Therefore, since the majority of reports of BP-based LIB/NIBs cycle the cells below the alloying potential (typically using a cutoff voltage of 0.02 V),^57,67,74,75^ BP anodes must undergo significant structural change. Indeed capacity deterioration has been shown to originate from the disintegration of electrodes and their delamination from current collectors by postcycling *ex situ* electron microscopy.^61^ However, there is still limited understanding of the physical and chemical manifestation of these structural and interphasial changes on BP, when cycled within operating cells.

In order to improve the performance of BP in alkali-ion batteries it is important to understand numerous electrode processes. From a structural perspective, characterizing particle expansion, cracking, strain, and crystal evolution is essential to optimizing performance parameters, such as rate and capacity, as well as suppressing capacity degradation and increasing cycle life.^76,77^ Whereas knowledge of interphasial process such as SEI formation and stability is key to managing processes including ion transport and irreversible capacity losses.^78^ Techniques including *in situ* transmission electron microscopy (TEM) have been used to study these phenomena,^57,76,79,60^ showing highly directional ionic transport properties. However, these experiments only approximately mimic real cells and utilize either zero electrolyte, with the alkali metal oxide on the Na or Li acting as a pseudo solid state electrolyte in an ultrahigh vacuum environment,^80,81^ or a thin-layer cell arrangement with a tiny volume of confined electrolyte.^82^ Furthermore, the energy of the high voltage electron beam can interfere with sensitive materials, causing side reactions that alter the results generated. Techniques such as *ex situ* X-ray photoelectron spectroscopy (XPS) have also been used to reveal the SEI composition at amorphous phosphorus electrodes,^83−85^ with one study on layered BP^69^ showing that P species are incorporated in the SEI and that severe electrolyte decomposition at the highly reactive surface of Na_3_P can be suppressed by the use of electrolyte additives, such as fluoroethylene carbonate (FEC). However, as with *ex situ* testing, the postcycling disassembly of cells and washing/drying of electrodes before analysis can induce electrode change and destruction.

Atomic force microscopy (AFM) is a powerful tool that can measure both the surface morphology and mechanical properties of electrode materials. This can be achieved *in situ* or *operando* in an electrochemical cell under operation (electrochemical-AFM (EC-AFM)), enabling real-time investigation of evolving electrode-electrolyte interphases during cycling. EC-AFM has been widely used to characterize next-generation electrode surfaces and has greatly impacted alkali-ion and other related battery research.^86^ Most existing studies have focused on graphite,^87−89^ Si,^90−93^ and metallic Li anodes,^94^ revealing factors such as the impact of electrolyte additives on the morphology and mechanical properties of the SEI.^86,89^ Although there are no EC-AFM studies of BP, MoS_2_, which is similarly layered and is known to undergo a conversion reaction at low voltages, has been shown to experience a large volume expansion.^95−97^ However, by observing the nucleation and growth of its ultrathin SEI with EC-AFM, it was established that an appropriate choice of electrolyte additive can protect the electrode from side reactions and reduce structural strains, which is an important step toward optimized MoS_2_-based electrodes.^95^

Using the *operando* EC-AFM we present an experimental study of the interphasial evolution of BP during sodiation under representative battery conditions. Electrode phenomena including: nucleation, growth, and evolution of the SEI; volumetric change upon intercalation; and electrode degradation/irreversible structural change upon alloying, have all been clearly distinguished and linked to specific electrochemical processes. Observations are corroborated with *ex situ* Raman spectroscopy and XPS, which correlate morphological changes to the evolving composition of the interphasial layers. These results provide significant insights into the crucial role both the SEI and electrode evolution play in the poor long-term performance, offering insights toward electrode engineering that can overcome these issues and enable the deployment of BP based anodes in high capacity alkali-ion batteries.

## Results and Discussion

### Electrochemical Characterization of Exfoliated BP Coin Cells

Before *operando* EC-AFM experiments were undertaken, the electrochemical performance of nanostructured BP was evaluated in Na-ion coin cells vs metallic Na in a 1 M sodium hexafluorophosphate (NaPF_6_) in ethylene/diethylene carbonate (EC/DEC) electrolyte. This electrolyte composition was chosen as the cyclability of Na–P binary compounds in Na cells can be substantially improved by the use of NaPF_6_ as an electrolyte salt, compared to sodium perchlorate (NaClO_4_).^98^

**Figure 2:**
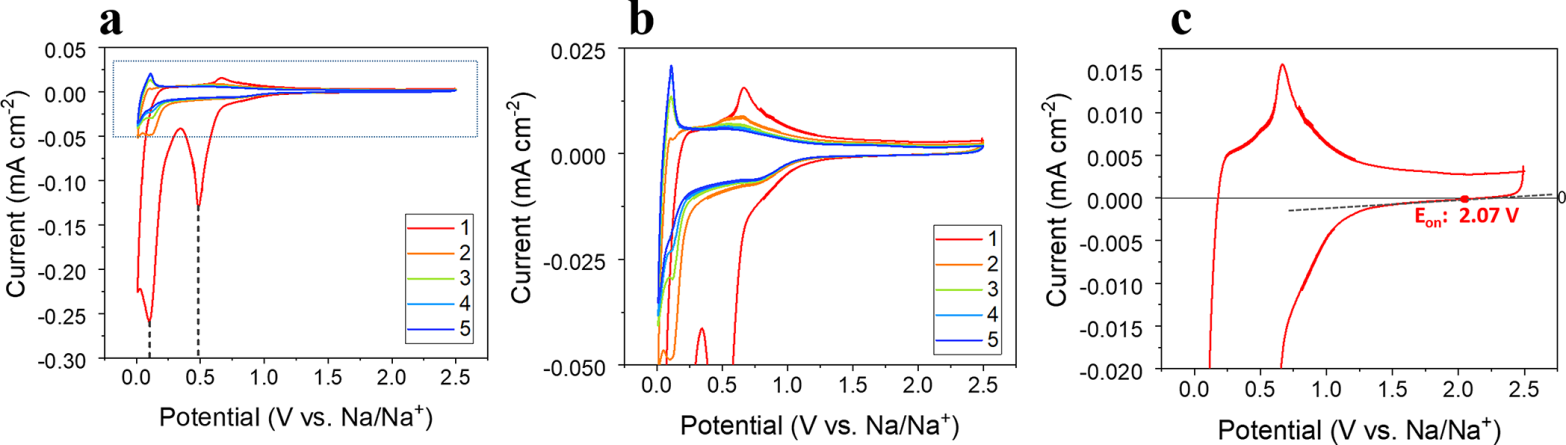
(a,b) CVs of the first 5 cycles. CVs of cycles 1–5 (red, orange, green, light blue, dark blue). (c) First cycle only, recorded from BP NIB coin cells at 0.1 mV s^–1^ with the electrolyte 1 M NaPF_6_ EC/DEC.

Figure 2 shows cyclic voltammograms (CVs) of the first five cycles for the BP electrodes at a scan rate of 0.1 mV s^–1^. During the first cathodic polarization the onset potential, calculated from interpolating the current back to zero (from the CV of the first cycle shown in Figure 2c), is measured as 2.07 V, below which point the current slowly grows to form a broad feature at ∼0.6 V with a major peak occurring between 0.8–1.2 V. This current response has been attributed primarily to the irreversible decomposition of the electrolyte to form an SEI layer on the surface of the BP anodes,^61^ although it will also encompass the response due to the intercalation of sodium between the BP layers (∼1.5–0.54 V).^57,60,69^ In the first cycles, two peaks also appear at ∼0.49 and ∼0.11 V (labeled with dashed gray lines in Figure 2a), which have been reported to relate to the alloying of Na with BP, theoretically resulting in Na_3_P (via *x*Na + P → Na_*x*_P(0.17 < *x* ≤ 3).^69,60^ In the reverse scan, three peaks were observed at 0.10, 0.51, and 0.69 V, related to the stepwise release of sodium ions from the sodiated BP, from Na_3_P to P (with an unknown structure), through an intermediate Na_*x*_P phase.^69^ In subsequent cycles, the cathodic peak at 0.49 V disappears, implying that the phosphorene layers in BP do not reform after the alloying reaction, and the other sodiation peak at 0.10 V is significantly reduced, which can be attributed to the loss of electrical contact of the active material from pulverization, as previously reported.^61^ On the contrary, the broad peak between 2.07 and ∼0.6 V remains (highlighted in the enlarged Figure 2b), as the volume expansion/contraction from the alloying reaction will likely expose “fresh” BP surface to the electrolyte, causing continuous regeneration of SEI.

To confirm the electrochemical sodium storage properties of BP, BP/Na half cells were evaluated by galvostatic charge–discharge measurements in the potential range of 2.5–0.01 V at 0.2 C (0.05 A g^–1^), as shown in Figure S1 (Supporting Information). The charge–discharge profiles of the 1st, 2nd, 3rd, 10th, and 50th cycles of the BP anodes are shown in Figure S1a (Supporting Information). The first discharge cycle shows four distinct electrochemical processes, with the first region between 2.5 and 0.6 V corresponding to the initial stages of the SEI formation that are responsible for the low first-cycle Coloumbic efficiency of ∼65% (Figure S1b, Supporting Information). The second slope between 1.2 and 0.7 V corresponds to the intercalation of sodium within BP layers, while the two final plateaus at ∼0.6 and ∼0.2 V relate to the different stages of alloying. In the subsequent cycle, the plateau at ∼0.6 V is no longer present, as the phosphorene layers do not reform after the alloying process. These results are consistent with the CV data in Figure 2 and literature reports for BP-based NIBs^57,65^ and LIBs.^56,99,100^ The specific capacity (calculated based on the mass of BP) and Coloumbic efficiency were measured over 70 cycles and are plotted in Figure S1b (Supporting Information). The specific reversible capacity of the BP after the first cycle was 1133 mAh g^–1^; however, after the 10th cycle, the reversible capacity significantly decreased by ∼50% to 534 mAh g^–1^, where it remained up to the 70th cycle (515 mAh g^–1^).

To further establish the relationship between electrochemical performance and electrode kinetics for BP, electrochemical impedance spectroscopy (EIS) measurements were performed. Figure S1c–e (Supporting Information) shows Nyquist plots of the BP anode at different states-of-charge in the first cycle, each comprising one semicircle in the high-medium frequency region and a straight line with two distinct angles at the medium-low frequency region. The semicircle corresponds to the interfacial resistance from the SEI surface film and the electrochemical charge transfer resistance between the active material and the electrolyte.^101,102^ The low-frequency straight line is attributed to sodium ion diffusion inside the active material.^65^ An equivalent circuit is schematically represented in Figure S3f.^101^

Figure S1f shows that at open-circuit voltage (OCV) the measured cell had a large resistance of ∼2.6 kΩ, and this gradually increased between OCV – 1.4 V, due to the generation of resistive SEI. During the intercalation stages, between 1.4–1.0 V, the subsequent charge transfer process increased the metallicity of BP, which in turn decreased the resistance. Finally, between 1.0–0.01 V the resistance dropped significantly to ∼37 Ω due to the formation of the fully sodiated and intermetallic Na_3_P phase.^103^ However, while the origins of the electrochemical signatures discussed above are widely reported, few studies actually directly link the electrochemistry to physicochemical change via *in situ* or *operando* experiments. This means the intricacies and interconnections between electrochemical, morphological and compositional change are largely unexplored.

### *Operando* EC-AFM Imaging of Interphasial Processes on Mechanically Exfoliated BP

**Figure 3:**
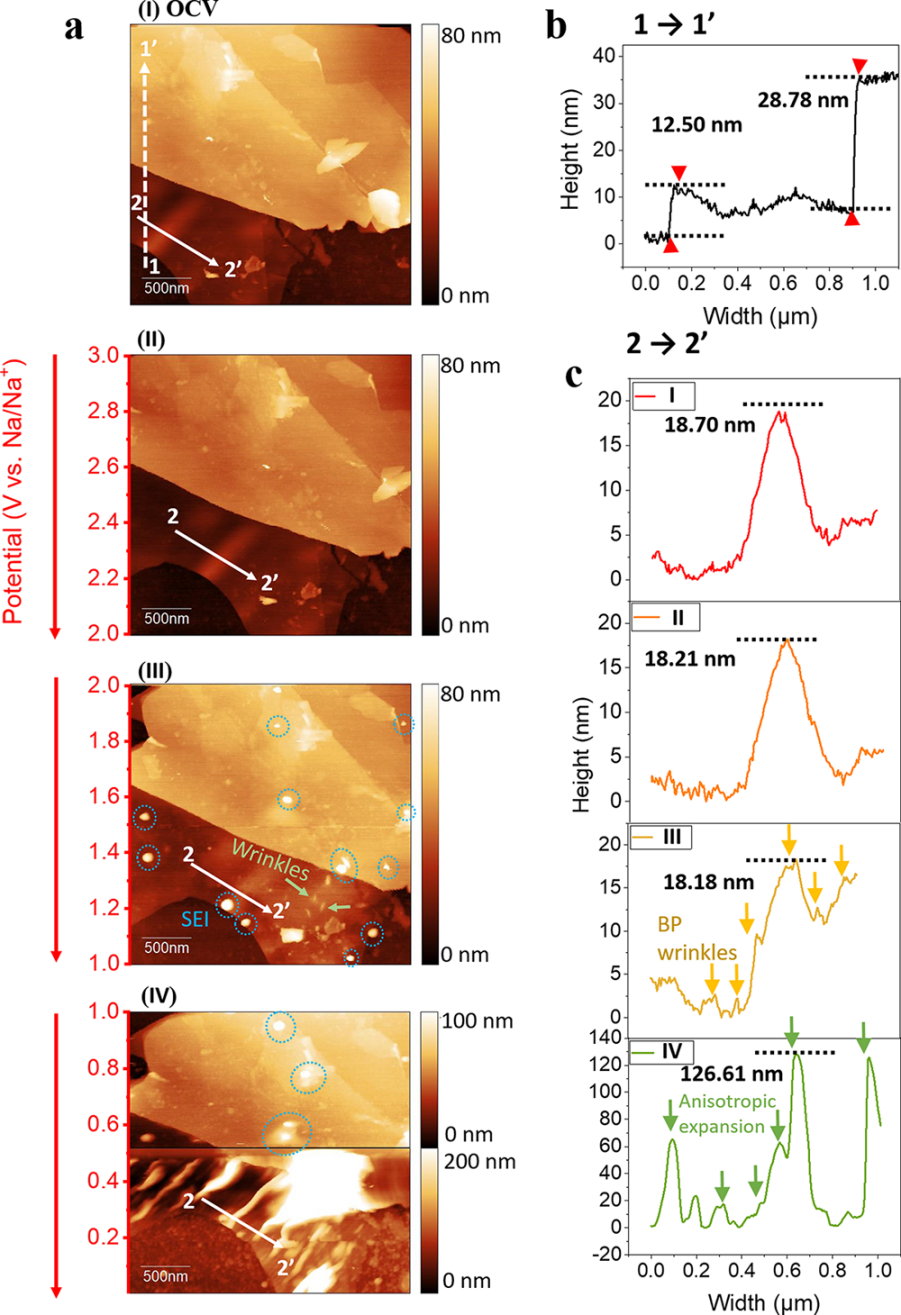
(a) Images of a 2.85 × 2.85 μm area on the surface of the BP anode in the electrochemical cell with 1 M NaPF_6_ EC/DEC electrolyte at (I) OCV, and (II–IV) *operando* EC-AFM images captured continuously in the range 3–0.01 V at 1 mV s^–1^, where each image captures 1 V. Scale bars are shown at the right side of the images, and corresponding voltages are quoted vs Na/Na^+^. (b) The height profile of the flake at OCV, taken across the dashed white line 1 → 1′. (c) Changes in the height profiles across the solid line 2 → 2′.

The ultra–flat surface of BP makes it an ideal substrate for characterizing dynamic electrode–electrolyte interphase processes including SEI formation and electrode evolution. Thin BP electrodes were prepared by mechanical exfoliation (schematic in Figure S2, Supporting Information) onto a Au current collector, forming a BP/Au electrode which was installed into the EC-AFM electrochemical cell illustrated in Figure S3 (Supporting Information). Figure 3a (I) shows the surface morphology of a typical flake of BP at OCV in a 1 M NaPF_6_ EC/DEC electrolyte. The flake was clean and flat, with well-defined terraces and steps. Line scans (across the area highlighted by the white dashed line) were used to determine the height profile of the BP steps, which is plotted in Figure 3b. It can be seen that the BP flake consisted of two large terraces with step edges of 12.50 and 28.78 nm in height, corresponding to ∼24 and ∼55 phosphorene layers respectively (∼0.52 nm per layer).^104^ An *ex situ* AFM image of the whole flake is shown in Figure S4 (Supporting Information).

*Operando* EC-AFM images were then taken of the BP surface while under electrochemical control vs Na. The CV from the BP/Au *operando* EC-AFM electrode cell can be found in Figure S5 (Supporting Information), where it is compared to an equivalent cell with excess BP, the bare Au current collector, and the BP anode coin cell. The CVs acquired with the AFM cell show the characteristic BP features at similar potentials to those in the coin cell, although due to the low BP loading the background contribution is more significant. The morphological changes of the BP electrode as a function of potential, in the range 3.0–0.01 V, are shown in the images in Figure 3b (II–IV) (images without annotation can be found in Figure S6, Supporting Information). The red arrow indicates the direction of scan, as each image dynamically captures 1 V. No significant changes are seen between the image captured at OCV in Figure 3a (I), and between 3–2 V in Figure 3a (II), consistent with the measured electrochemical response shown in Figure 2 and Figure S5e (Supporting Information). Interestingly, below 2 V, the growth of a small number of discrete nanoparticles on the basal plane can be observed (highlighted in blue dotted circles), which is consistent with the kinetically favorable, low overpotential growth of SEI at defects, as observed for SEI at graphite step edges in LIBs,^105^ or similar to the behavior observed when metal nanoparticles are electrodeposited onto carbonaceous materials.^106^ This assessment is supported by the increased density of these SEI particles as the potential approaches ∼1 V, where the SEI derived current response becomes more dominant. Other origins of the particles are also possible, however, including the development of sodium clusters, although the deposition voltage vs Na/Na^+^ and the measured mechanical properties of the particles (using the reduced Young’s modulus calculated according to the Derjaguin–Muller–Toporov (DMT) model − explained in the (Supporting Information)) during imaging (Figure S7 (Supporting Information)), lead us to believe this is less likely. The average basal plane roughness, measured above the alloying potential, increases from 0.63 ± 0.09 nm in Figure 3a (III), to 1.70 ± 0.21 nm in Figure 3a (IV) owing to the subsequent spreading of SEI across the BP surface. It is apparent that the SEI film is relatively thin and uniform across both basal planes and edge sites, in contrast to observations at graphitic anodes, where SEI has been observed to be significantly thicker at step edges where it accumulates.^89^

Below 1.4 V, sodiation is accompanied by the propagation of wrinkles across the lower BP basal plane, highlighted with green arrows in Figure 3a (III). Nanowrinkles are distinguished from nanoparticles by their long cylindrical shape and unidirectional alignment, caused by the development of folds in the structure of the BP, as is highlighted in Figure S8 (Supporting Information). This phenomenon has been reported previously for studies of ultrathin BP with *in situ* TEM and *ex situ* XRD,^57^ as well for MoS_2_ nanosheets.^95,96^ Intercalation into the “channels” within the BP crystal has been shown to result in compressive stresses that are mitigated by the flexibility of the longer P–P bonds, leading to mechanic strains that manifest as linear distortions.^104^ To quantitatively characterize these structural changes, the height profile across the bottom terrace during the cathodic scan was measured, following the line indicated by the white solid arrows 2 → 2′. These line scans are plotted in Figure 3c (I–IV), confirming the presence of nanofolds as peaks and valleys particularly, as shown in Figure 3c (III), due to the intercalation of sodium. This is consistent with previous reports whereby the intercalation of both sodium and lithium lead to charge stripes/doping in the zigzag crystallographic direction.^60,104^ The height of these nanofolds were extrapolated from this line profile to be on average ∼2 nm on the BP plane (Figure 3c (III)).

Further sodiation below 0.52 V results in the formation of Na_*x*_P species (via an alloying reaction), with a consequent anisotropic volume expansion of ∼580%, calculated from the maximum peak heights between Figure 3c (I) and (IV), which is consistent with previous *in situ* TEM and *ex situ* XRD reports, where an approximately 500% volume expansion was reported.^57^ Interestingly, these long and thin wrinkles follow the same parallel orientation as the wrinkles initially propagated at higher potentials (1.4 V in Figure 3a (III)) from the directional sodiation of BP into the “channels”.^104,60^ Figure S9 (Supporting Information) shows another case where multiple sodiation-induced stripes can be observed across the Na intercalated BP flake, likely resulting from a large anisotropic expansion in the zigzag orientation. This gradual increase of the electrode surface area leads to continuous regeneration of a freshly exposed BP surface in contact with the electrolyte, therefore driving the further formation of SEI, contributing to irreversible capacity losses (seen in Figure 2 and Figure S1 (Supporting Information)). Subsequent contraction during the desodiation of Na_*x*_P (at 0.10, 0.51, and 0.69 V from Figure 2) is responsible for the structural fracturing of the secondary electrode structure and the mechanical detachment and breakdown of the SEI, which additionally limits cycling stability of BP electrodes.^60,107^ These effects are further highlighted with *in situ* AFM in Figure S10 (Supporting Information) which shows that the final structure of BP is significantly compromised, even after one cycle.

Although the underlying Au current collector can be observed to undergo some interphasial change during cycling (Figure 3, Figure S9, Figure S11, and Figure S12 (Supporting Information)), this is found via XPS to be primarily due to the formation of SEI (below ∼2.0 V). Some Na–Au alloying will also occur below ∼0.7 V (Figure S11 (Supporting Information)), as discussed in the literature,^108,109^ possibly followed by Na plating close to 0 V. However, as each of these reactions rely on the close contact of Na ions with the Au, the BP layer sitting flat on the metallic surface will largely passivate the area of gold underneath it (Na ions cannot diffuse through the BP plane). Hence, surface change of the supporting gold is not expected to strongly influence the BP behavior observed.

Nevertheless, the electrochemical sodiation of Ag, Cu and Si, in comparison to Au, were tested in order to confirm it as the most suitable current collector for studying BP (Figure S13 (Supporting Information)). While Au was shown to present the least surface changes (Figure S13a, Supporting Information), demonstrating why it has been used in prior studies of the BP interface,^57,76,110^ the behavior of BP during sodiation on an Ag substrate was also investigated as Ag does not alloy with Na. *In situ* images acquired across four individual flakes during cycling, to avoid any possibility of tip-induced disruption to the BP, are presented in Figure S14 (Supporting Information). These are consistent with BP/Au data, demonstrating that an inhomogeneous SEI develops on the surface of BP during the cathodic sweep from OCV to 1.4 V, followed by the anisotropic wrinkling of BP between 1.4 and 1.2 V, and finally the large volume expansion derived disintegration of BP from the alloying reaction between 0.6–0.2 V.

**Figure 4:**
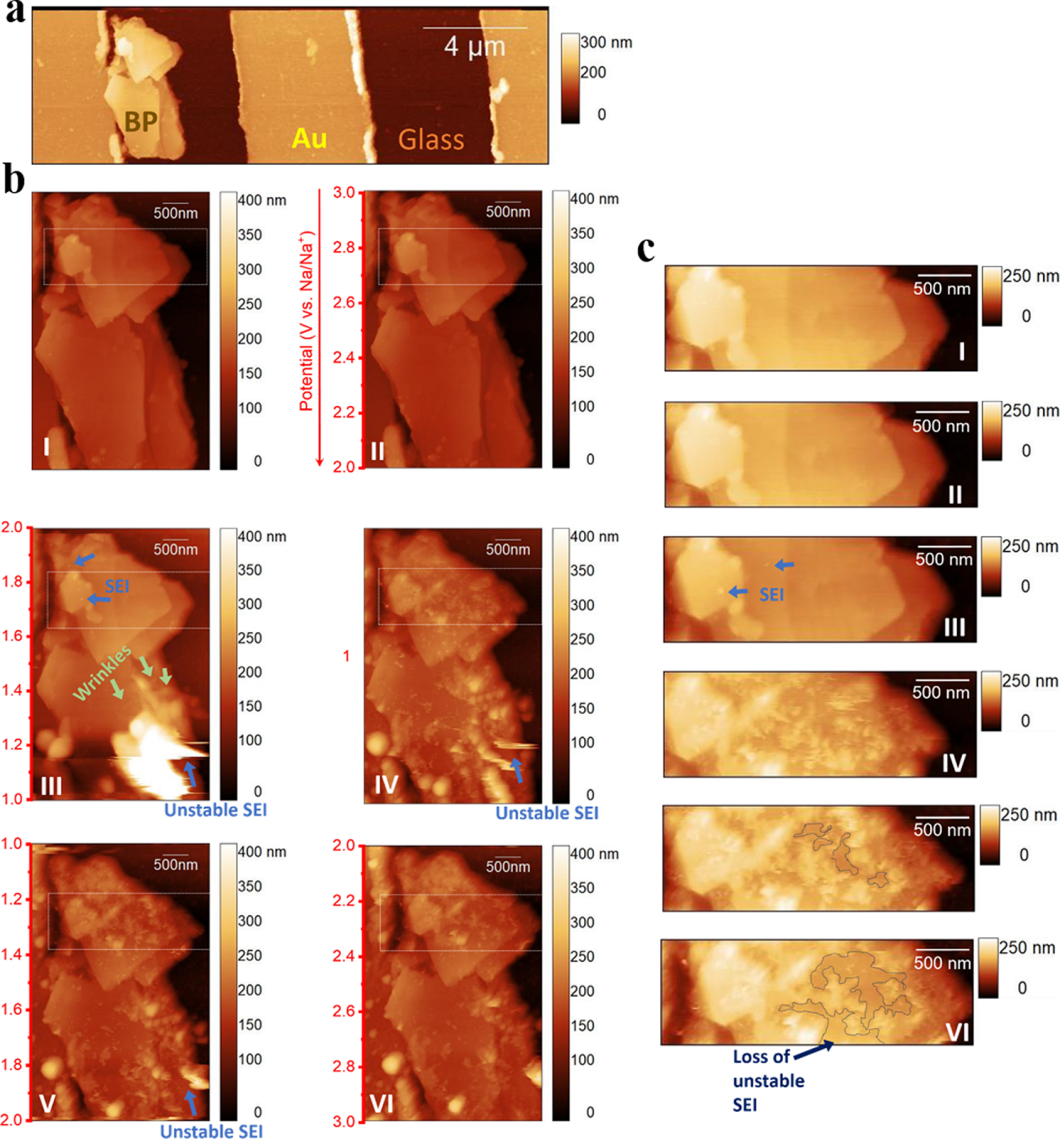
(a) AFM image of an *ex situ* 20 × 6 μm area scan of BP flakes on an Au/Glass current collector substrate. (b (I)) *Operando* EC-AFM images across 10 × 4 μm area on BP/Au/Glass surface in 1 M NaPF_6_ EC/DEC electrolyte at OCV, and (II – III) captured continuously during the cathodic scan (3–1 V) at 0.5 mV s^–1^. Each image captures 1 V and scale bars are shown at the right side of the images. (IV) The same flake is captured as the voltage was held at 1 V, and in (IV–VI) during the reverse anodic scan (1–3 V). All corresponding voltages are quoted vs Na/Na^+^. (c) Enlarged images of the flake edges taken from the dotted white lined box in (b), with the white line representing 500 nm.

Finally, to further exclude possible underlying substrate effects, studies were undertaken using Au/glass interdigitated electrodes (Figure 4), where the BP was supported by inert glass. An *ex situ* AFM image is presented in Figure 4a, showing an exfoliated BP flake that is electrically connected to Au, but lying on-top of the inert glass substrate.

As discussed above, the composition, morphology, or even the onset potential for SEI formation on BP are all important factors to understand if irreversible losses are to be minimized, and all are still areas of debate in literature.^57,66,79,111^ In order to allow deeper investigation of the initial stages of growth and nature of the SEI formed at BP, *operando* experiments were performed within a restricted voltage window (3–1 V) to avoid any influence of alloying-induced material change.

Figure 4b (I–VI) shows a series of *operando* images of SEI formation and sodiation of pristine BP flakes, consistent with the data in Figure 3 (images without annotation can be found in Figure S15 (Supporting Information)). Indicated by the blue arrows, the deposition of small SEI particles is again found to begin close to 2 V, and below 1.4 V the BP flake starts to wrinkle from the early stages of intercalation of sodium within the BP layers (highlighted by green arrows), but here this is accompanied by a large and unstable accumulation of SEI below 1.38 V (Figure 4b (III)). This unstable deposit is mobile under the tip, as indicated by its eventual detachment, thus reducing the resolution in the affected area. The interphasial (SEI) instability is further demonstrated in Figure 4c (I–VI) which magnifies the morphological change in the region outlined with a dashed box in Figure 4b (I–VI). In this series of images, the initial nucleation of SEI particles can be clearly observed, as highlighted with the blue circles during the cathodic scan (Figure 4c (III)). The mechanical properties of these particles were also characterized (Figure S7 (Supporting Information)). From Figure S7d (II) (Supporting Information), the Young’s modulus of the initial SEI particles grown was measured at ∼4.68 GPa, significantly lower than the value expected from Na metal^112^ but in line with that measured for SEI structures.^89^ During a subsequent scan, while the electrode was held at the 1.0 V cutoff voltage (Figure 4c (IV)), these SEI nanoparticles can be seen to have accumulated to form a distinct thin film at the BP electrode–electrolyte interphase, forming a soft layer with a modulus measured at ∼3.0 GPa (calculated from Figure S7e (II) (Supporting Information)). However, in the reverse anodic scan (Figure 4c (V–VI)), a large fraction of the SEI layer formed has dissolved into the electrolyte or detached from the BP, leaving only part of the surface passivated. This therefore presents a site for future SEI accumulation.

It is apparent from these data (and that presented in Figure 3, and Figure S7 (Supporting Information)) that the SEI film that forms on BP is thin and has relatively uniform Young’s modulus across both basal planes and edge sites. This is very different to the SEI that forms at graphitic anodes, where SEI has been observed to be significantly thicker and softer at step edges where it accumulates,^89^ but is consistent with findings for MoS_2_.^95,113^ We hypothesize that this this related to the unidirectional diffusion pathway of Na into BP, as a result of its anisotropic crystal structure. For BP, the diffusion barrier for Na across in the zigzag direction (across the basal plane) is significantly lower than that found for other layered materials (0.04 eV)^60^ leading to an ultrahigh diffusivity, 10^4^ times faster than that across the graphene basal plane.^114^ This increased flux would drive SEI species to accumulate at the surface rather than step edges.^115^

### Characterizing the Chemical and Structural Composition of the BP/SEI Interphase

**Figure 5:**
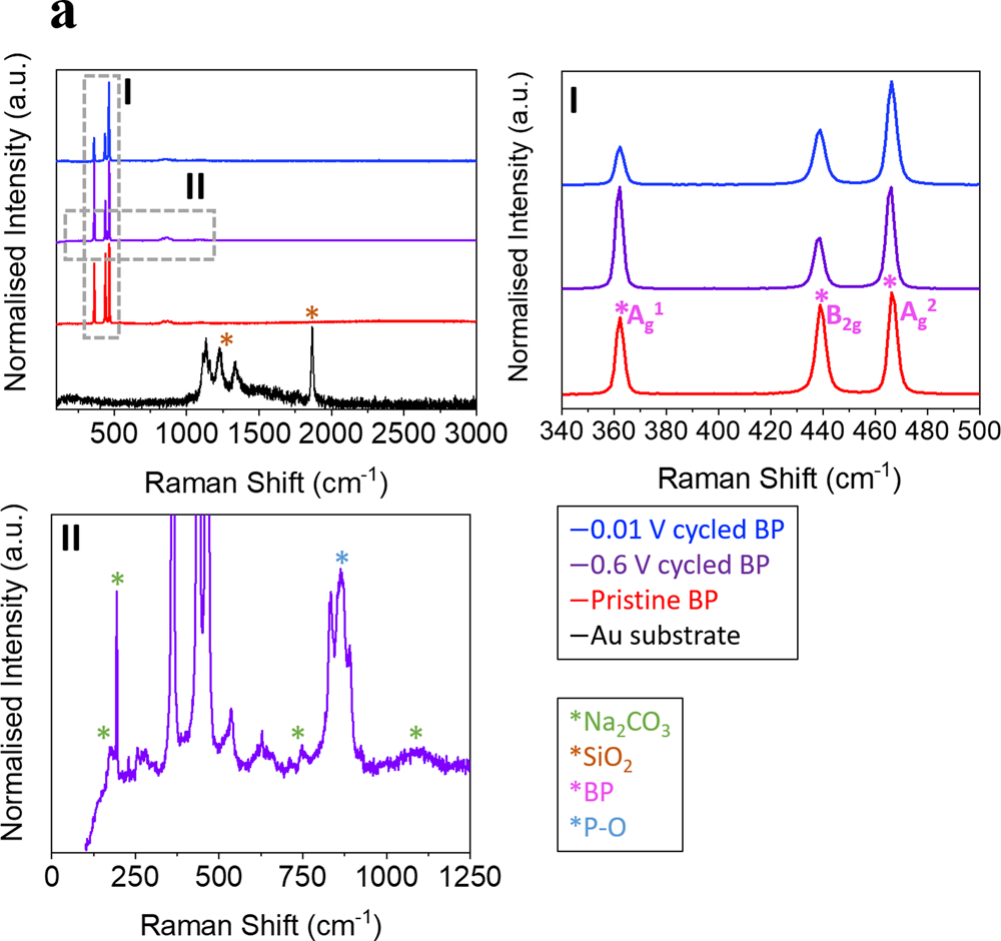
(a) Comparison of the representative Raman spectral intensities acquired for the pristine substrate (Au/quartz (SiO_2_)), a substrate deposited with BP, and the BP deposits cycled in the range 3–0.6 V (vs Na^/^Na^+^). Raman shifts are shown across 100–3000 cm^–1^ to show full range of intensities and zooms are shown in (I) across the Raman shift range of 340–3500 cm^–1^ to highlight peak intensities corresponding to the BP and in (II) the range of 0–1250 cm^–1^ for the discharged BP electrode cycled between 2.5–0.6 V to highlight the additional peaks present.

*Ex situ* Raman spectroscopy was used to determine the influence of cycling depth on the crystallinity of the BP electrode and the chemical composition of the SEI layer formed at the interphase (Figure 5). The Raman spectra of BP after charging and discharging within prealloying potentials (2.5–0.6 V) and postalloying potential (2.5–0.01 V) were collected and compared with those from pristinely exfoliated electrodes (Figure 5a). BP has three characteristic Raman active modes, A_g_^1^, B_2g_ and A_g_^2^, corresponding to the out-of-plane vibration (∼361 cm^–1^) and the in-plane vibrations along the zigzag (∼438 cm^–1^) and armchair (∼466 cm^–1^) directions, respectively.^116^ The vibrational modes are represented in the schematic in Figure S16b (Supporting Information). A downshift in all the peak positions indicates residual charging of the layers, while the full width half-maximum (FWHM) gives an indication of the homogeneity of the structure.^103^Figure 5a (I) shows the three characteristic Raman modes of BP, which can be observed in both pristine and cycled samples. This indicates that the BP layers are partially maintained or reformed after one cycle, consistent with the literature.^57^ The corresponding peak positions and FWHM values were measured and are presented in Table S1 (Supporting Information). To calculate this, three Lorentzian components were fitted as shown in Figure S16 (Supporting Information). No significant change in the peak position of all modes can be observed across the different electrodes, as there is no remaining charge to the layers following the discharging anodic sweep. However, the FWHM of all the reformed BP peaks increases, by 26, 37, and 34% for the A_g_^1^, B_2g_ and A_g_^2^ respectively, when charged and discharged down to alloying potentials. The increased width following charging results from a decrease in the phonon lifetime due to increased disorder induced scattering. On the other hand, the FWHM for all BP peaks remains relatively constant for the electrode cycled above the alloying cutoff window. The lack of broadening in this case indicates no disordering is induced and thus that the intercalation part of the sodiation is completely reversible. These results are consistent with the EC-AFM images depicting the almost complete breakdown of the BP electrodes post full cell cycling.

The chemical composition of the SEI layer formed on the discharged electrodes can also be characterized with Raman spectroscopy. When the BP electrode was cycled to 0.6 V and analyzed in the discharged state, a series of new peaks appeared in the Raman spectrum, at 182, 194, 228, 257, 228, 628, 706, 748, 863, and 878 cm^–1^, along with a broad peak at ∼1090 cm^–1^. Sodium carbonate, Na_2_CO_3_, is responsible for the features at 182, 194, 228, (corresponding to T (Na, CO_3_)), and 706.0 cm^–1^ (corresponding to the **(**CO_3_)^2–^ ν_4_ doubly degenerate asymmetric bend), while the broad peak at ∼1090 cm^–1^ likely encompasses the known positions for Na_2_CO_3_ at 1079 and 1083 cm^–1^ (corresponding to the **(**CO_3_)^2–^ ν_1_ nondegenerate symmetric stretch), as well as some influence from the underlying Au/quartz substrate.^117−119^ The peaks between ∼800 and 880 cm^–1^, present in all BP samples (also shown in Figure S17 (Supporting Information)), result from oxidized P–O.^120^ The measured Raman signals and their attributions are summarized in Table S2. These Raman features can be directly linked to the SEI, supported by the EC-AFM data in Figure 3, Figure 4 and Figure S7. However, the specific composition of the SEI cannot be determined from this data due to the similarity in functional groups of the possible reduction products of EC, including sodium ethylene dicarbonate ([CH_2_OCO_2_Na]_2_), sodium butylene carbonate ([CH_2_CH_2_OCO_2_Na]_2_) and sodium carboxylate (RCOONa).^121^ The relative lack of SEI related peaks in the BP electrode cycled down to 0.01 V (other than those reported for Na_2_CO_3_ shown in Figure S17 (Supporting Information)), further confirms that the SEI layer is unstable, becoming partially removed as a result of the structural expansion from the alloying reaction of BP with alkali-ions.

**Figure 6:**
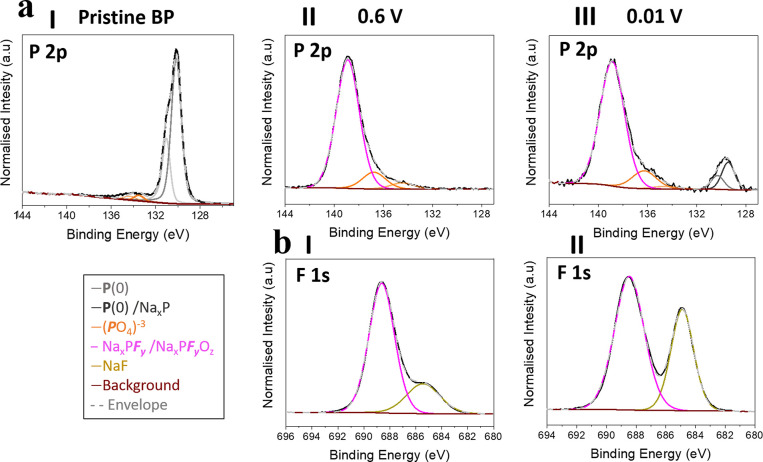
(a) High-resolution P 2p XPS spectra for pristine BP (I), BP charged and discharged between 2.5–0.6 V (II), and 2.5–0.01 V (III), respectively. (b) F 1s spectra of the BP electrode charged and discharged in the same 2.5–0.6 V (I), and 2.5–0.01 V (II) ranges.

To further elucidate the correlation between the Na^+^ ion storage mechanisms and the interphasial properties for BP electrodes, the chemistry of the SEI was investigated using XPS without any air exposure. Figure 6 and Figure S18 (Supporting Information) highlight the differences in SEI chemical composition for BP electrodes in the discharged state, after they had been cycled between 2.5 and 0.6 V and between 2.5 and 0.01 V, and show a comparison to “freshly” exfoliated pristine BP. The electrodes were carefully removed from the EC-AFM cell air free, rinsed in DEC, and finally dried under vacuum overnight prior to analysis. The corresponding relative elemental compositions are compared to pristine bulk BP electrodes in Table S3 (Supporting Information).

The P 2p spectra are compared in Figure 6a (I – III). In the precycling pristine BP spectrum (Figure 6a (I)), two clear peaks are observed: elemental phosphorus P(0) at 130.1 eV, and a small phosphate contribution (PO_4_^3–^) at 134.0 eV, indicating partial surface oxidation of BP which is unsurprising due to its significant oxygen sensitivity.^105^ After electrochemical cycling to 0.6 V (Figure 6a (II)) a new peak at ∼138.8 eV can be seen to develop from the presence of fluorinated phosphorus species, such as Na_*x*_PF_*y*_O_*z*_ and/or Na_*x*_PF_*y*_, suggesting the formation of a surface layer originating from the reductive decomposition of PF_6_^–^ anions.^122^ Additionally, a lower intensity peak at 133.2 eV, could be assigned to monovalent-phosphorus (P^+^) species, such as NaH_2_PO_2_ or PR_2_(OR′), which have been reported as a component of the SEI layer for BP anodes in NIBs with the same electrolyte composition.^69^ Finally, the loss of the elemental P(0) peak implies the formation of a significant surface layer, such as the one shown in Figure 4, as XPS is a surface sensitive technique.^123^ After the BP was cycled down to alloying potentials (from 2.5 V down to 0.01 V) and analyzed in the discharged state (Figure 6b (III)), a peak at 129.4 eV can be detected. This feature could arise from the return of the elemental P(0) peak, suggesting SEI layer species have been partially removed to reveal fresh BP, as has been seen in the data from Figure 4. However, since it is shifted to a lower energy than that of elemental phosphorus (130.2 eV), it is likely to also encompass a contribution from the reduction product of P, Na_*x*_P. The presence of this binary compound suggests that electrically isolated material is generated from the significant morphological disruption after alloying, which is in good agreement with the EC-AFM and Raman data.^83,69^

Figure S18 (Supporting Information) shows the C 1s, O 1s, and Na 1s spectra for the electrodes cycled to 0.6 V (Figure S18a (Supporting Information)) and 0.01 V (Figure S18b (Supporting Information)) respectively. The primary components of the SEI layer are determined to be organic species such as ROCO_2_Na, CH_3_ONa, (CH_2_OCO_2_Na)_2_, and inorganic species including Na_*x*_PF_*y*_ and/or Na_*x*_PF_*y*_O_*z*_, NaF and Na_2_CO_3_.^124−131^ The results are consistent with similar reduction products for both Na and Li electrolytes. Related comparisons of the SEI for LIBs and NIBs has been recently reported.^132^ The F 1s spectral peaks, in Figure 6b (I and II), further confirm that the SEI is partly composed of inorganic components. The peaks at 688 eV are likely to derive from fluorinated phosphorus species, Na_*x*_PF_*y*_ and/or Na_*x*_PF_*y*_O_*z*_, and those at 685.3 eV from NaF. Interestingly, the signal intensity of the NaF species increases with deeper electrochemical cycling. Since it is generally accepted in literature that SEI is composed primarily of an inner layer of inorganic compounds and an outer layer of organic species,^133^ the increase in intensity of the NaF could arise from a combined effect, where highly reactive Na_*x*_P compounds more favorably reduce the electrolyte, and that expansion from the alloying displaces the underlying inorganic SEI layers. These results, in addition to the EC-AFM and Raman data presented, shed light on the inability of the SEI layer to withstand the significant structural changes that are accompanied by the disintegration of active BP material during alloying. This suggests that the traditional methods for controlling or tuning SEI properties with electrolyte additives,^70^ or nanosizing and hybridizing with carbon supports,^57−68^ may not be enough to overcome these limitations and that further consideration into the fundamental alkali-ion storage mechanism must be taken when designing new BP based anodes for NIBs and LIBs.

## Conclusion

Through a combination of *operando* EC-AFM and *ex situ* spectroscopy, this study provides a fundamental understanding of the modes of morphological, mechanical, and chemical change in layered intercalation-alloying alkali battery materials under operating conditions. Using the sodiation of BP as an exemplar system, the crucial potential-dependent structure–activity relationships in this material class are visually determined at the nanoscale. The insights gained can now inform the design of electrode structures, additives or system controls to counter, contain, or alleviate the mechanisms of degradation and failure that hold back these high capacity materials.

In particular, this work reveals the characteristic formation and evolution mechanisms of the SEI layer at BP, showing that interphasial species nucleate at a high onset potential and accumulate to form a thin yet unstable SEI, even when the cutoff voltage is restricted to the intercalation region. Furthermore, the severe material consequences that derive from BP sodiation are visualized, where anisotropic nanowrinkles propagate due to stresses associated with the intercalation of sodium ions through the BP “channels”. These wrinkles then grow larger into parallel stripes, causing a detrimental volume expansion before the layered morphology completely disintegrates upon alloying. This demonstrates that the primary methods currently used to pacify intercalation-alloying materials in batteries, e.g., nanosizing, carbon supporting, or hybridization, are unlikely to be sufficient to stabilize BP or similar anode materials when used in isolation. Further research into alternative modification strategies, such as the use of protective surface coatings to prevent direct contact between electrode/electrolyte, or the appropriate choice of electrolyte composition and film forming additives is needed, only then will BP based batteries reach viability.

## Methods

### CV Tests of Electrochemical Cells and Coin Cells

Macroscopic crystals of BP (99.998% purity) from SmartElements were used to make all electrodes. The CR-2032 coin cells were constructed from exfoliated BP working electrodes (45% BP, 45% carbon black and 10% polyvinylidene fluoride (PVDF – Solef 5130) on copper foil) with sodium metal counter electrode (Alfa Aesar 99.95% (metals basis) and a polypropylene separator (Celgard, 9 mm diameter). For these coin cells, BP was exfoliated via a liquid-phase exfoliation method adapted from a procedure reported previously.^57^ BP was dispersed in N-methyl-2-pyrrolidone, anhydrous (NMP – 99.8% (Merck)) (cylindrical vial, 20 mL NMP) at a concentration of 0.1 mg mL^–1^ in an argon filled glovebox. The vials were sealed, removed from the glovebox, and sonicated in an ultrasonic bath (Ultrawave QS3, 50 W) for 12 h with the bath water changed every 20 min in order to keep the water temperature below 40 °C. The resultant dispersion was transferred back to the glovebox and into a sealed Buchi vessel (B-585 Drying). The solution containing Buchi was left under vacuum and heated at 80°C for 1 week to evaporate the majority of the NMP. The residual filtrate was then scraped into a cylindrical vial and placed in a glass-metal transition tube where it was evacuated further to <10^–6^ mbar using a turbomolecular pump and left under dynamic vacuum (continuous pumping) for 1 week, before the temperature was increased to 100 °C for a further week, leaving behind a powder of exfoliated BP. A typical slurry was made from carbon black (EQ-Lib-Super C45, MTI Corp) and the BP powder and PVDF binder in a mass ratio of 45:45:10, mixed in NMP manually via pestle and mortar. The mass loading of active material (BP) was ∼0.460 mg cm^–2^, corresponding to a total mass loading of ∼1.01 mg cm^–2^ and a thickness of ∼6.5 μm. CV measurements were made using a Gamry Interface 1000 potentiostat.

Additional electrochemical data (charge–discharge and EIS) were collected using a Gamry Interface 1000 potentiostat within the potential range of 0.02–2.5 V(versus Na/Na^+^) at 0.2 C (0.05 A g^–1^), with the BP/Na CR-2032 coin cells as assembled as above. The specific capacity was calculated based on the weight of phosphorus. For EIS tests, the coin cells were discharged–charged between OCV and 0.01 V for 1 cycle, with a constant current density of 0.2 C (0.05 A g^–1^). EIS were taken at potentials of 2.0, 1.6, 1.5, 1.4, to 1.0, and 0.01 V. The potentiostatic EIS test was set from a frequency of 100 kHz to 0.01 Hz, at an AC voltage of 10 mV. The Nyquist plots of the EIS were obtained and fitted with Gamry Echem Analyst.

For the AFM cell, BP was mechanically exfoliated via the “scotch tape” method^134,135^ onto the substrates to make the electrodes (see schematic in Figure S2 (Supporting Information)). The substrates used were Au sputtered quartz (Pi-kem, polished quartz wafer sputtered with a 10 nm Ti adhesion layer followed by 30 nm of Au) and Au/glass interdigitated electrodes (Metrohm DropSens). The electrode area was defined using an adhesive polyimide film (kapton) punched with a 5 mm diameter hole. The counter/reference electrode was a Ni wire wrapped with sodium foil, which was placed near the working electrode inside the electrolyte (1 M NaPF_6_ in EC/DEC (1/1 (v/v), FluoroChem). A schematic of the EC-AFM can be seen in Figure S3 (Supporting Information).

### Structural Characterization

*Operando* EC-AFM (Bruker Dimension Icon with ScanAsyst) experiments were carried out in an Ar-filled glovebox (Mbraun YKG series) with H_2_O < 0.1 ppm, O_2_ < 0.1 ppm combined with a CH Instruments electrochemical workstation (Model 700E Series Bipotentiostat). The film morphology was characterized using PeakForce tapping mode with a RTESPA-300 silicon probe with a reflective Al coating (Bruker Corp., k = 40 N m^–1^, f_0_ = 300 kHz). All of the results obtained from the AFM were analyzed by Gwydion software. PeakForce tapping mode was utilized to image the electrodes in fluid. In this mode the cantilever oscillates, far below the resonant frequency, and the vertical motion of the cantilever using the main piezo element (Z) relies on the feedback force. The real feedback loop maintains a constant maximum interaction force (peak force) between the probe and the sample surface at each pixel, in order to obtain topography of that sample. This method can provide atomic level resolution at low imaging forces, preserving the sample and tip, enabling imaging of the delicate soft interphasial layers with high accuracy. This mode also reduces interference during liquid phase imaging, compared to tapping mode as it does not require the probe to oscillate at resonance frequency.^136^ The Young’s modulus was calculated using the same QNM PeakForce tapping mode with the RTESPA-525 silicon probes with reflective Al coating (Bruker Corp., k = 200 N m^–1^, f_0_ = 525 kHz) and using the relative method to calibrate against HOPG (18 GPa). Further details about the model used can be found in the Supporting Information.

After the electrochemical tests, the BP electrodes were taken from the EC-AFM cell and rinsed with DEC to remove any residual electrolyte salt, followed by drying for 24 h in the glovebox at ambient temperature. The Raman spectra were collected using a Renishaw In-Via microscope equipped with a 785 nm laser through a 20× objective. To keep the samples air free, the electrodes were contained in argon environment and loaded in a custom-made glass cell.

### Spectroscopic Characterization

Raman spectra were collected using a Renishaw In-Via microscope equipped with a 785 nm laser through a 20× objective. To keep the samples air free, the electrodes were contained in argon environment and loaded in a custom-made glass cell. Surface analysis was also carried out with X-ray photoelectron spectroscopy (Thermo Scientific Kα). The spectra were collected at room temperature using monochromaric Al–Kα (1486.6 eV) radiation as an incident X-ray source. The electrodes were placed on a sample holder with carbon conductive tape in an argon-filled glovebox. The sample holder was introduced into a load-lock chamber using a transfer vessel (Thermo Scientific 831-57-100-2) without air exposure.

## Supporting Information Available

Additional experimental details, including materials preparation and *operando* EC-AFM cell schematics, and additional supporting charge–discharge, EIS, AFM, Raman and XPS data (PDF)
