# Supplementary material for: Black Phosphorus Degradation during Intercalation and Alloying in Batteries
Source: ACS Nano. 2023 Mar 27;17(7):6220–33. doi: 10.1021/acsnano.2c08776 (PMC10100570; doi:10.1021/acsnano.2c08776)
Supplement: Supplementary file 1 — nn2c08776_si_001.pdf [file nn2c08776_si_001.pdf]

Supporting Information

Black Phosphorus Degradation during Intercalation and Alloying in Batteries.

*Samia Said[1], Zhenyu Zhang[1], Rebecca R. C. Shutt[3], Hector J. Lancaster[3], Dan J.L. Brett,[1], Christopher A. Howard[3], Thomas S. Miller[1]. **

[1]Electrochemical Innovation Lab, Department of Chemical Engineering, University College London, Torrington Place, London,

UK, WC1E 7JE

[2]The Faraday Institution, Quad One, Becquerel Avenue, Harwell Campus, Didcot,

UK, OX11 ORA

[3]Department of Physics & Astronomy, University College London, Gower Street, London,

UK, WC1E 6BT

Electrochemical characterization of exfoliated BP coin cells.

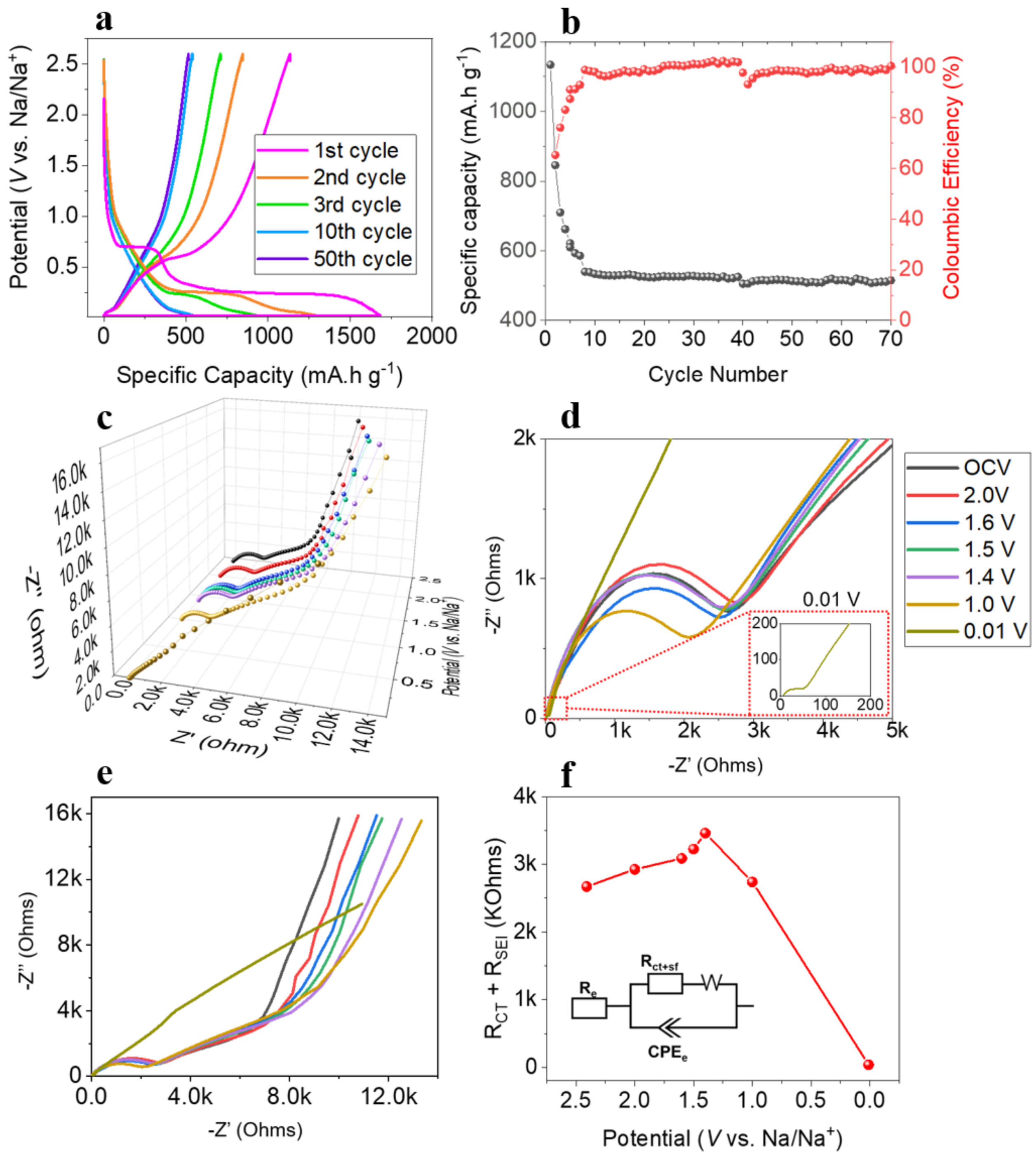

Figure S1. (a) The charge–discharge profiles of the BP electrodes during the 1st, 2nd, 3rd, 10th and 50th between 2.5 – 0.02 V with a current density of 0.2 C. (b) The cycling performance and Coulombic efficiency of the BP electrodes at a current density of 0.2 C. (c−e) The *in situ* Nyquist plots of electrochemical impedance spectroscopy (EIS) measurements in the

discharge–charge process, at different discharging potentials. (f) Sum of resistance of SEI film and charge transfer during the first discharge–charge (with schematic showing equivalent circuit model in the bottom left corner).

Mechanical exfoliation of BP.

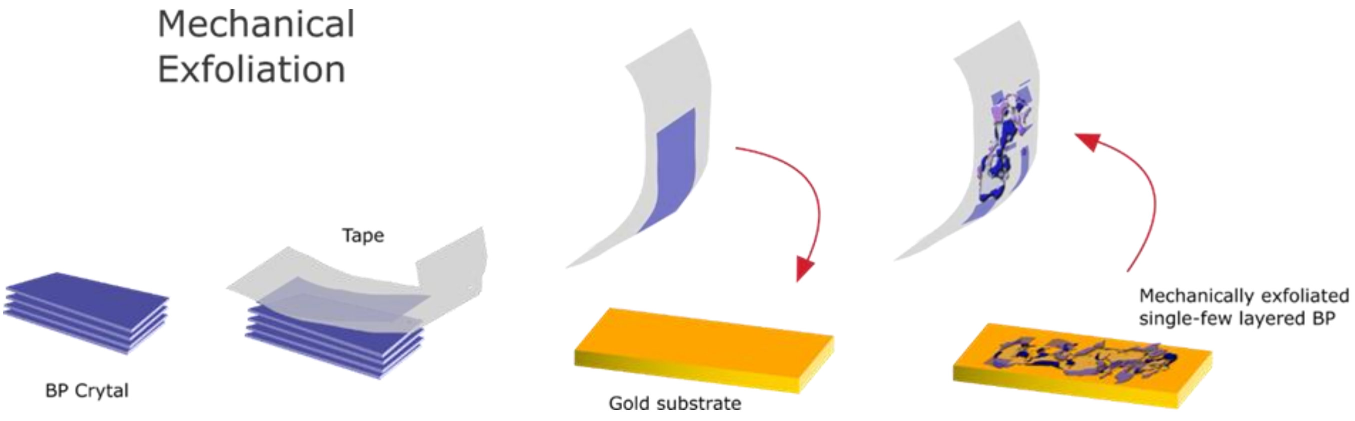

Figure S2: Schematic of mechanical exfoliation of BP onto Au sputtered quartz crystal electrodes via the scotch tape.

Na EC−AFM cell.

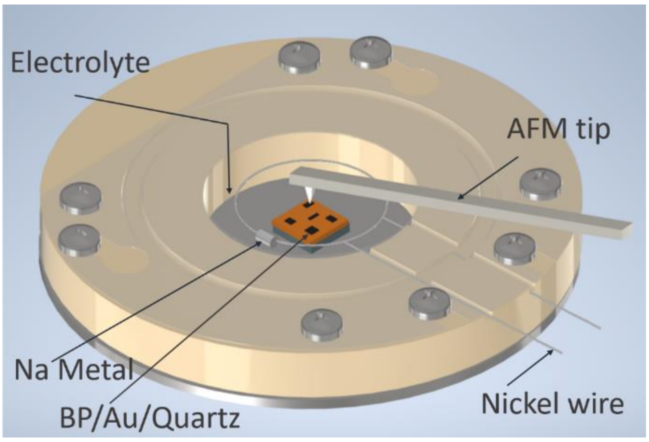

Figure S3. A shematic of the EC-AFM cell, with the working electrode as exfoliated BP on Au sputtered quartz crystal. The counter and reference electrode is an Na metal wrapped Nickel wire. The electrolyte sits in the opening, that is sealed with an o-Ring and open glass window.

Ex situ Characterisation of exfoliated BP with AFM.

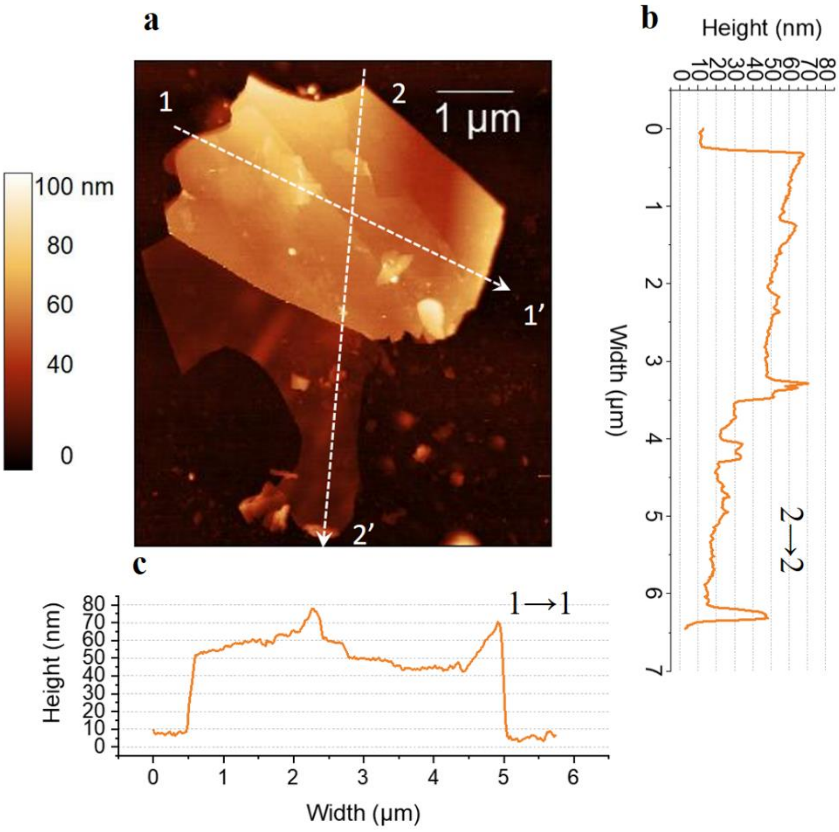

Figure S4. (a) *Ex situ* AFM image of BP flake on Au substrate with an area of 5 x 6 µm (1 µm scale bar is top the left of the image) The height profiles plotted in (b) and (c) from line scan analysis across the 2→2’ and 1→1’ dotted white arrows respectively.

Characterizing the electrochemistry of BP: AFM cell vs. coin cell.

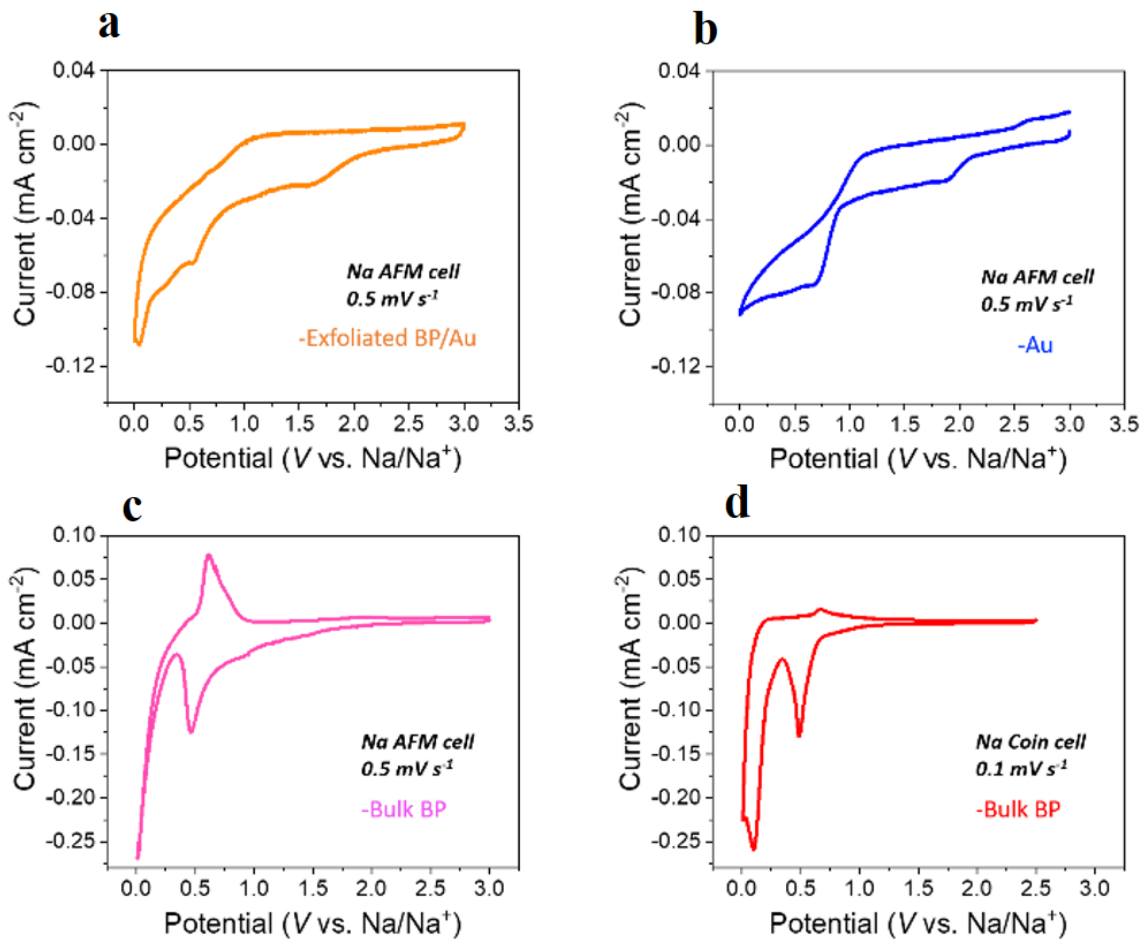

Figure S5. A comparison of the first cyclic voltammetry (CV) curves for the Na EC-AFM cell with (a) exfoliated BP/Au electrode, (b) Au bare electrode and (c) the covered BP/Au electrode, in 1M $NaPF_6$ EC/DEC at 0.5 mV s^{-1}. (d) Represents the first CV of the BP electrodes in a BP/Na coin cell at 0.1 mV s^{-1}, cycled in the same electrolyte composition. All corresponding voltages are plotted vs. Na/Na^+.

Figure S5 presents a comparison of the electrochemical response from the first cycle of a BP/Au electrode, with the bare Au electrode in the EC-AFM coin cell is made, to distinguish between electrochemical responses from BP vs. Au. Given the low material loading of exfoliated BP/Au electrode, the current density in Figure S5a is largly dominated by the supporting Au (Figure S5b), although a slight additional peaks do develop at 0.49 V and 0.11 V fom the alloying.

When the Au is completely covered with BP in the Na EC-AFM cell in Figure S5c, the electrochemistry largly resembles that of the bulk BP Na ion coin cell. In both cases, the onset potential is found to be ~2.07 V, which is consistent with the morphological changes on the surface of BP seen repeatedly in Figure 3, Figure 4, Figure S9 and Figure S14 in the range ~2 to 0.6 V. This therefore can soley be attributed to SEI formation on the surface of BP.

***Operando* EC-AFM imaging of interfacial processes on mechanically exfoliated BP.**

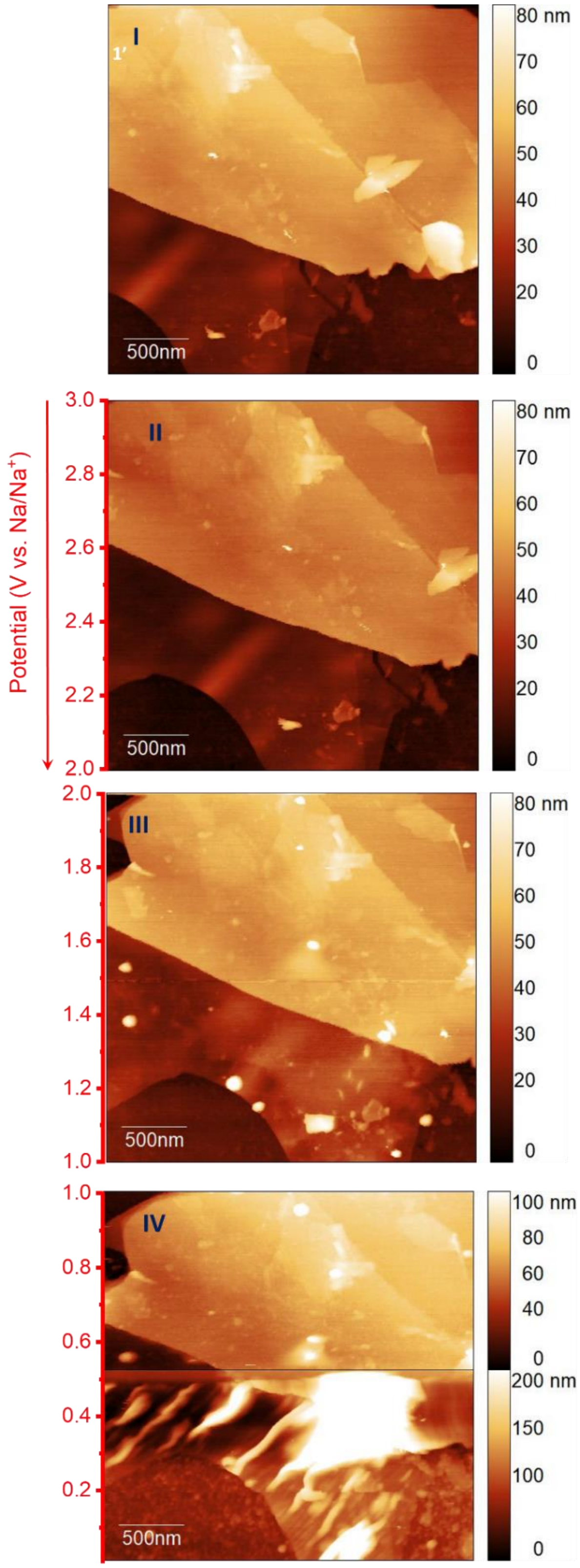

Figure S6. Un annotated of 2.85 × 2.85 μm area on the surface of the BP anode in the electrochemical cell with 1M $NaPF_6$ EC/DEC electrolyte at (I) open circuit voltage (OCV), and (II−IV) *operando* EC-AFM images captured continuously in the range 3 − 0.01 V, where each image captures 1 V and scale bars are shown at the right side of the images. All corresponding voltages are quoted vs Na/Na^+.

Measuring the Young's modulus of the SEI formed on exfoliated BP.

PeakForce tapping mode is an extension of the tapping mode that can probe the sample and measure the pN-level interaction forces directly from the deflection of the cantilever.[1] The cantilever oscillates, but far below the resonant frequency, and the vertical motion of the cantilever using the main piezo element (Z) relies on the feedback force. The real feedback loop maintains a low force interaction between the tip and sample at 10 pN at actuation rates of up to 8 Hz in air or fluid. This method can provide atomic level resolution at low imaging forces, preserving the sample and tip, enabling imaging of the most delicate of samples with high accuracy.

Before each experiment, the probe was calibrated using the relative method by using a standard HOPG (modulus = 18 GPa) sample in Ar atmosphere for a precise measurement of mechanical properties. Through which, the deflection sensitivity, tip radius and spring constant values were recorded. Nano-indentation mechanical measurements were conducted at the same time as the morphology was mapped. By recording the load and displacement of the specialized tips and cantilevers when being pressed into the surface, a force-distance curve is generated, which is further used to calculate the hardness, elastic modulus and various viscoelastic properties of the materials.

An important parameter calculated from the force-distance curve is the Young's modulus, by fitting the retracting curve with the Derjaguin-Muller-Toporov (DMT) model. The DMT theory of adhesion describes the adhesion between hard elastic solids with long range adhesive interactions.[1] The DMT model used to estimate the sample's reduced elastic modulus from the forces taking place during the tip-sample interaction are described as.[2]

$$F_{interaction} = \frac{4}{3}E^{*}\sqrt{R}(d - d_0)^3 + F_{adhesion}$$

[1]

Where $F_{interaction}$ is the tip-sample force, E^{*} is the reduced elastic modulus of the tip, R is the radius of the tip, $d_0$is the surface rest position, $(d - d_0)$ is the sample deformation, and is the adhesion force during the contact. The sample's Young's modulus, E_s, can be derived from the reduced Young's modulus by the following Equation:[3]:

$$E^{*} = \left[\frac{1 - {v_t}^2}{E_t} + \frac{1 - {v_s}^2}{E_s}\right]^{-1}$$

[2]

Where E_t is the Young's modulus of the conical indenter (i.e. the tip) and v_t is the Poisson's ratio of the conical indenter (i.e. the tip), and v_s is the Poisson's ratio of the sample. If the sample modulus is much larger than that of the tip E_s > E_t, then the following applies;

$$E^{*} = \frac{E_s}{1 - {v_s}^2}$$

[3]

The DMT model works well for hard samples that are analysed with a very sharp tip. There are two calibration methods used to gather quantitative information. The calibration method used herein is known as the relative method. It uses a sample of known modulus to obtain the ratio of spring constant to the square root of tip end radius, since according to the DMT model:

$$E^{*} \propto \frac{(F_{interaction} - F_{adhesion})}{\sqrt{R}(d - d_0)^3}$$

[4]

Where:

$F_{interaction} = k \times \Delta z,$

[5]

And k is the spring constant of the tip, and Δz is the distance moved by the tip in the vertical direction. Therefore, in substituting the above, it can be seen that;

$$E \propto \frac{k}{\sqrt{R}}$$

[6]

The ratio of the spring constant and radius are therefore adjusted as to get the correct modulus of the known reference sample. Once this is carried out, the Young's modulus of unknown samples can be determined, relative to the reference sample.

In this work RTESPA-525 silicon probes with reflective Al coating (Bruker Corp., k = 200 N m^{-1}, f_0 = 525 kHz). All the results obtained by EC-AFM were analysed by Nanoscope Analysis software.

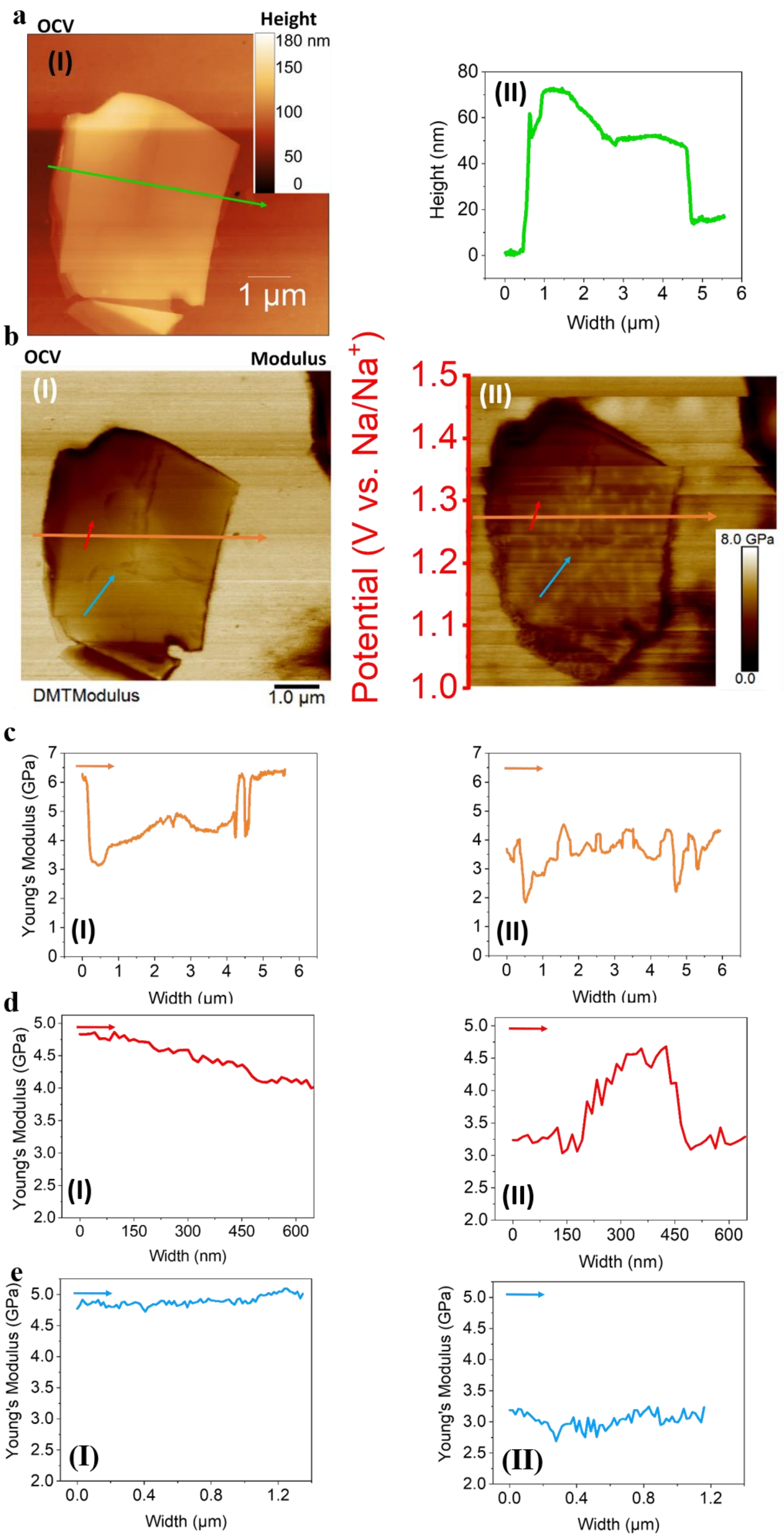

a
OCV
Height
(I)
180 nm
150
100
50
0
1 μm
(II)
Height (nm)
Width (μm)
b
OCV
Modulus
DMTModulus
1.0 μm
Potential (V vs. Na/Na+)
1.5
1.4
1.3
1.2
1.1
1.0
8.0 GPa
0.0
c
Young's Modulus (GPa)
Width (um)
d
Width (nm)
e
Width (μm)

Figure S7: *Operando* EC-AFM height images captured continuously from the electrochemical cell with the 1 M $NaPF_6$ EC/EMC electrolyte in a 7 × 7 μm^2 area on the surface of the BP/Glassy carbon anode (0.5 V per capture, at 0.5 mV s^{-1}). The signals of height at OCV (a (I)), the line scan analysis of the profile along the green line is shown in (a (II)), and the DMT modulus (b) are presented. All corresponding scale bars are shown at the right side of the images. All voltages are quoted vs Na/Na^+. (c−e) line scan analysis of the height profile recorded across orange (c), red (d), and blue (e) taken from the arrows in (b).

A good SEI should be flexible enough to survive the volumetric changes of the electrode while still maintaining a strong contact with the electrode surface. Hence, characterizing the mechanical properties of the SEI layer is important to develop high performance electrolyte–electrode systems for NIBs. Figure S7 presents a series of *operando* EC-AFM images of Bulk BP/Glassy carbon anode with the EC/DEC electrolyte discharging from 3.0 V (OCV) to 1.0 V. Each image was captured over 0.5 V. Height (a) The Young's modulus (b) (calculated according to the DMT model, explained in above) are presented within a square scan area of 7 × 7 μm.

The first column of images (Figure S7a−b (I)) displays the morphology and mechanical properties of the initial BP surface at OCV. The freshly exfoliated BP has a very clean and smooth surface after it is submerged into the electrolyte. The flake height measured at 473 nm which consists of ~910 BP layers.[4] The DMT modulus is recorded along each line scan shown in Figure S7 and the average Young's modulus quoted are calculated by roughness analysis across the whole BP basal plane for each image. The initial average Young's modulus of BP is measured to be 4.8 ± 0.67 GPa, with small fractures/plane edges on the surface plane, resulting in a lower Young's modulus of ~4.4 GPa. From ~1.5 V to ~0.5 V (Figure S7b (II)), the measured modulus decreased to an average of 3.3 ± 0.30 GPa, as small particles begin to appear

on the surface. Importantly, the modulus value is inhomogeneous across the BP surface, showing regions of lower modulus (~3.0 GPa averaged from the blue line profile shown in Figure S7e (II), as well as particles exhibiting a stronger modulus (~4.68 GPa calculated from the maximum point in the red line profile shown in Figure S7d (II). This is concurrent with the cathodic current increase, which can be attributed to the early stages of electrolyte decomposition. These early stage decomposition products typically consist of softer intermediate radical anion near the BP surface, (consistent with the lower interfacial layers grown onto graphite), [5] as well as the growth of inorganic species such as NaF, Na_2O, and Na_2CO_3, which are dense, thin, and relatively stable species, more similarly consistent with lower SEI layers of Si.[6]

Glassy carbon is chosen as the support in this case as the surface remains consistent down to 0.2 V, therefore BP SEI morphology and mechanical properties are clearly captured without substrate disruption, however glassy carbon has sodium storage capacities,[7] and therefore is not investigated further.

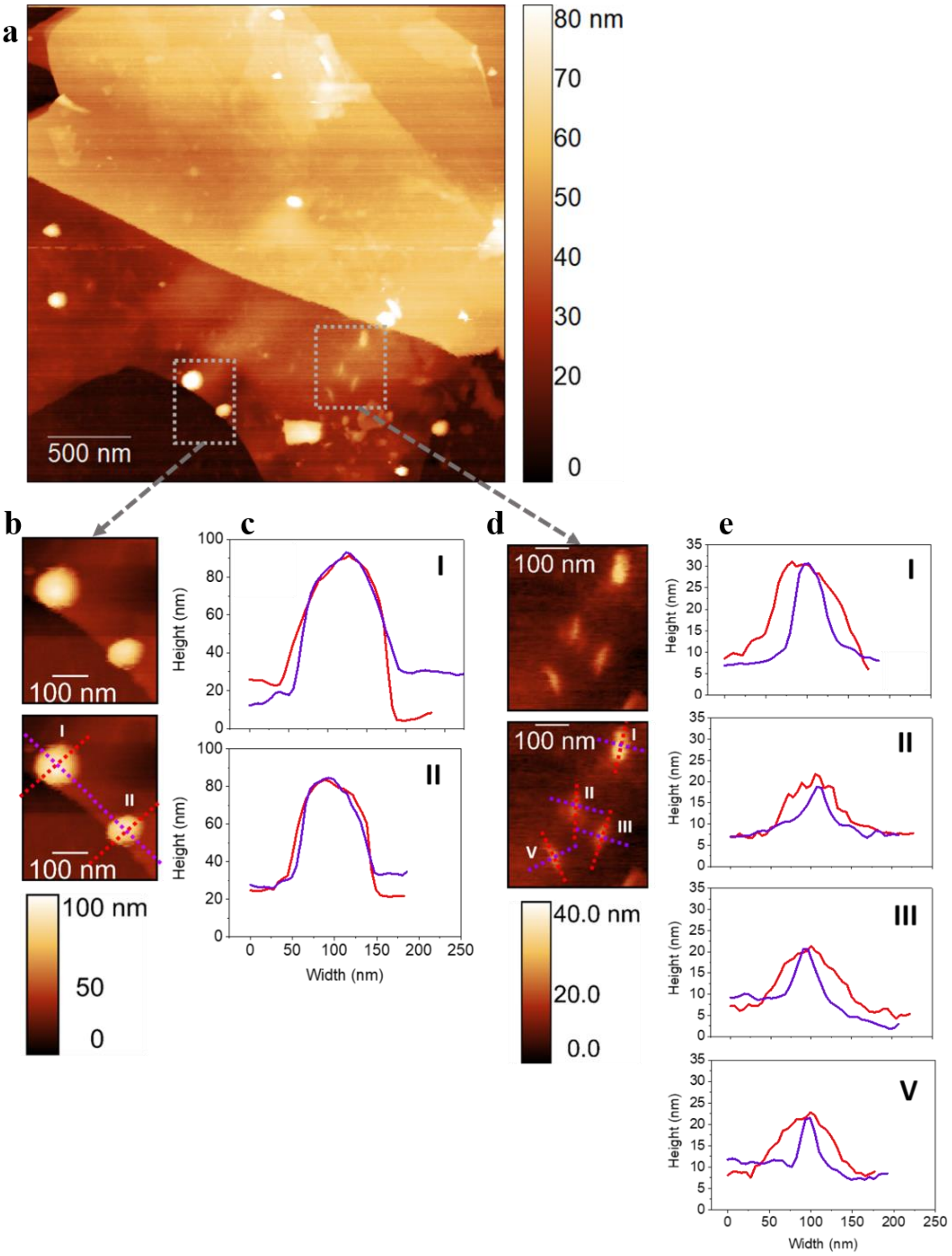

Figure S8: (a) 2.85 × 2.85 µm area on the surface of the BP anode in the electrochemical cell with 1M $NaPF_6$ EC/DEC electrolyte during *operando* EC-AFM imaging from 3 ─ 2 V. (b) Cropped image (460 × 490 nm) of the nano-particles, and (d) the nano-wrinkles (400 × 470 nm). Scale bars for all images are shown in the corner of the images. (c) and (e) show line scan analysis of the length and width versus height profiles for nano-particles and nano-

wrinkles respectively. The length is labelled by the red dotted lines and the width is labelled by the purple dotted lines.

A comparison of the shapes of SEI nano-particles and intercalation nano-wrinkles is represented in Figure S8. Line scan analysis of the length and width versus height profiles for nano-wrinkles and nano-particles taken from Figure S8a. Figure S8c (I−II) shows the height profiles taken across the nano-particles labelled in Figure S8b (I−II), whilst Figure S8e (I−V) shows the height profiles taken across the nano-wrinkles labelled in Figure S8d (I−V). From Figure S8e (I−V), it can be seen that the length (labelled by the red dotted lines) across all nano-wrinkles is ~50% larger than the width (labelled by the purple dotted lines). On the other hand, Figure S8c (I−II), shows that the length (labelled by the red dotted lines) across all nano-particles is approximately the size as the width (labelled by the purple dotted lines).

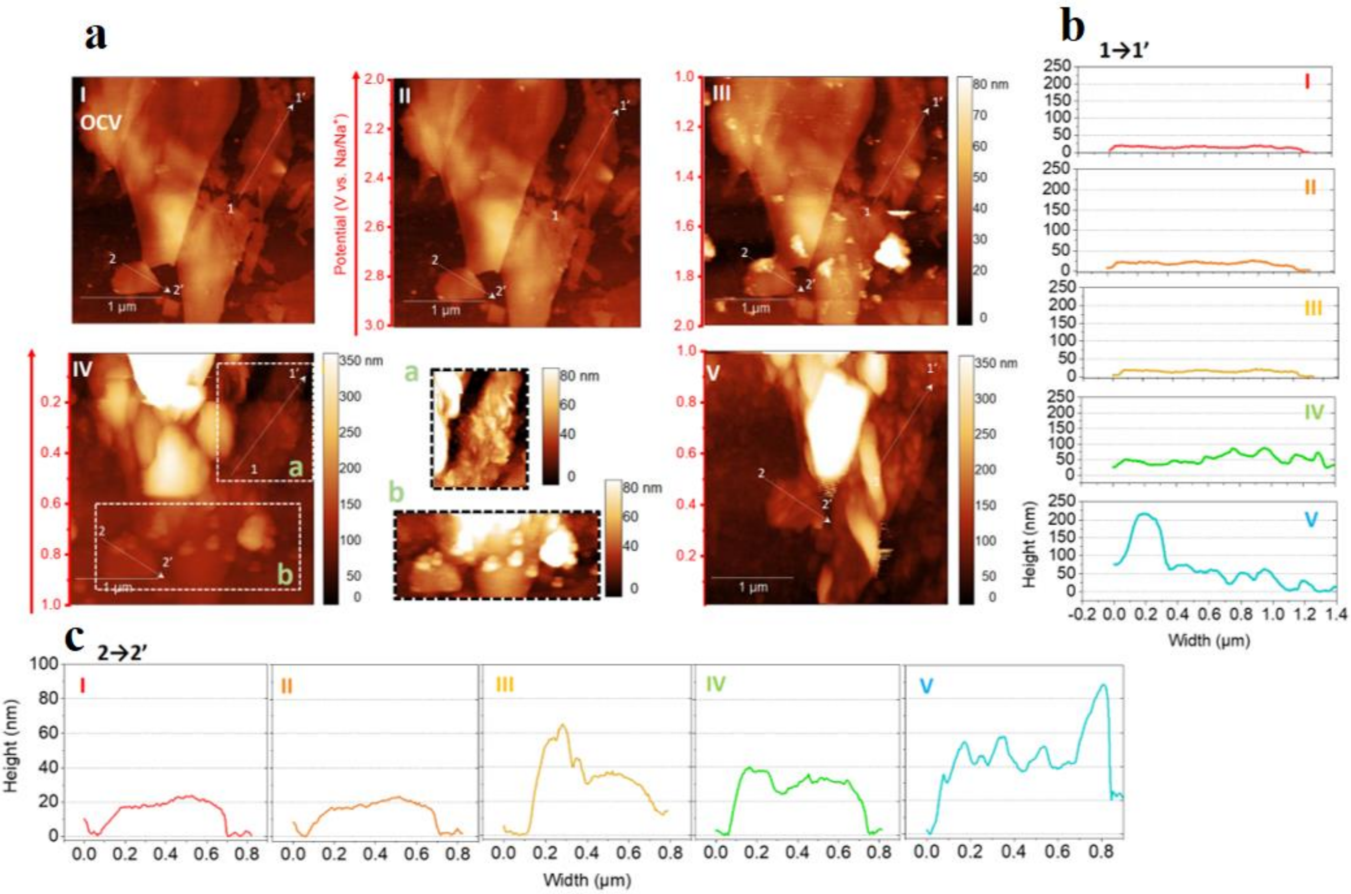

Figure S9. (a) *Operando* EC-AFM images of BP flake on Au substrate in 1 M $NaPF_6$ EC/DEC electrolyte in a 2.85 x 2.85 µm at, (I) OCV, then (II – V) scanning in the range 3 ─ 0.01 V. (IV a─b) enlarged images of (IV) the area covered by the green dotted box, to highlight height changes. All scale bars are to the right of the image, and all voltages plotted vs. Na/Na^+. (b) Line scan of BP flake during sodiation plotted as the height profiles taken across 1→1’ and, (c) taken across 2→2’ white dotted lines.

***In situ* EC-AFM imaging of BP after one charge/discharge cycle.**

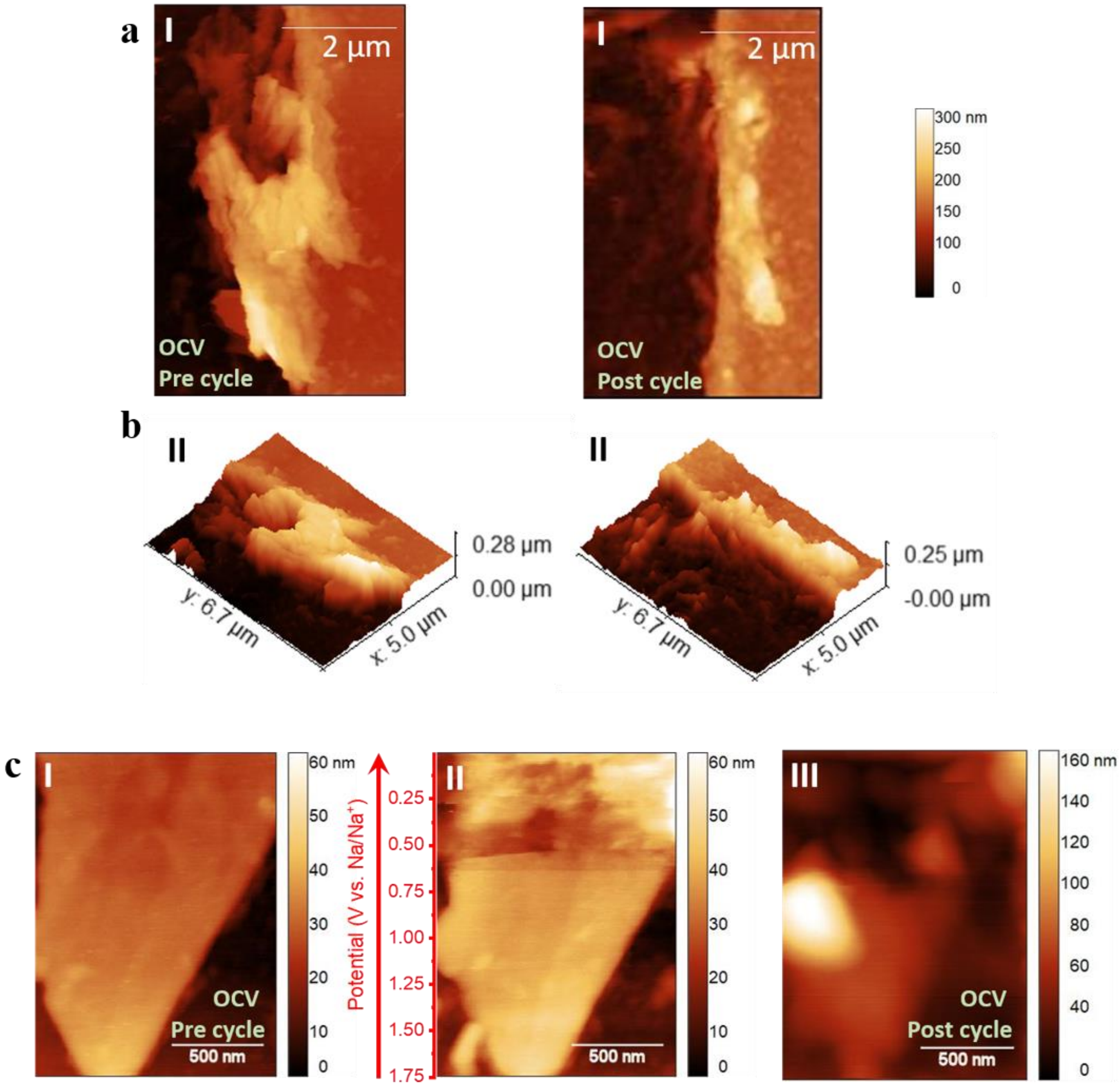

Figure S10. (a) *In situ* EC-AFM images of a BP flake on the Au/Glass electrode across 5 x 6.7 µm in 1 M $NaPF_6$ EC/DEC electrolyte area at (I) OCV (II), and post cycling from 3 ─ 0.01 V. (b) The 3D representations of (a). (c) Another BP flake on Au substrate across 2 x 1.5 µm area

at (I) OCV, (II) during cycling from 1.75 ─ 0.01 V with the red arrow indicating the direction of the scan, and (III) OCV post charge/discharge cycle between 3 ─ 0.01 V.

A **background study.**

Operando **EC-AFM imaging of interfacial processes on Au.**

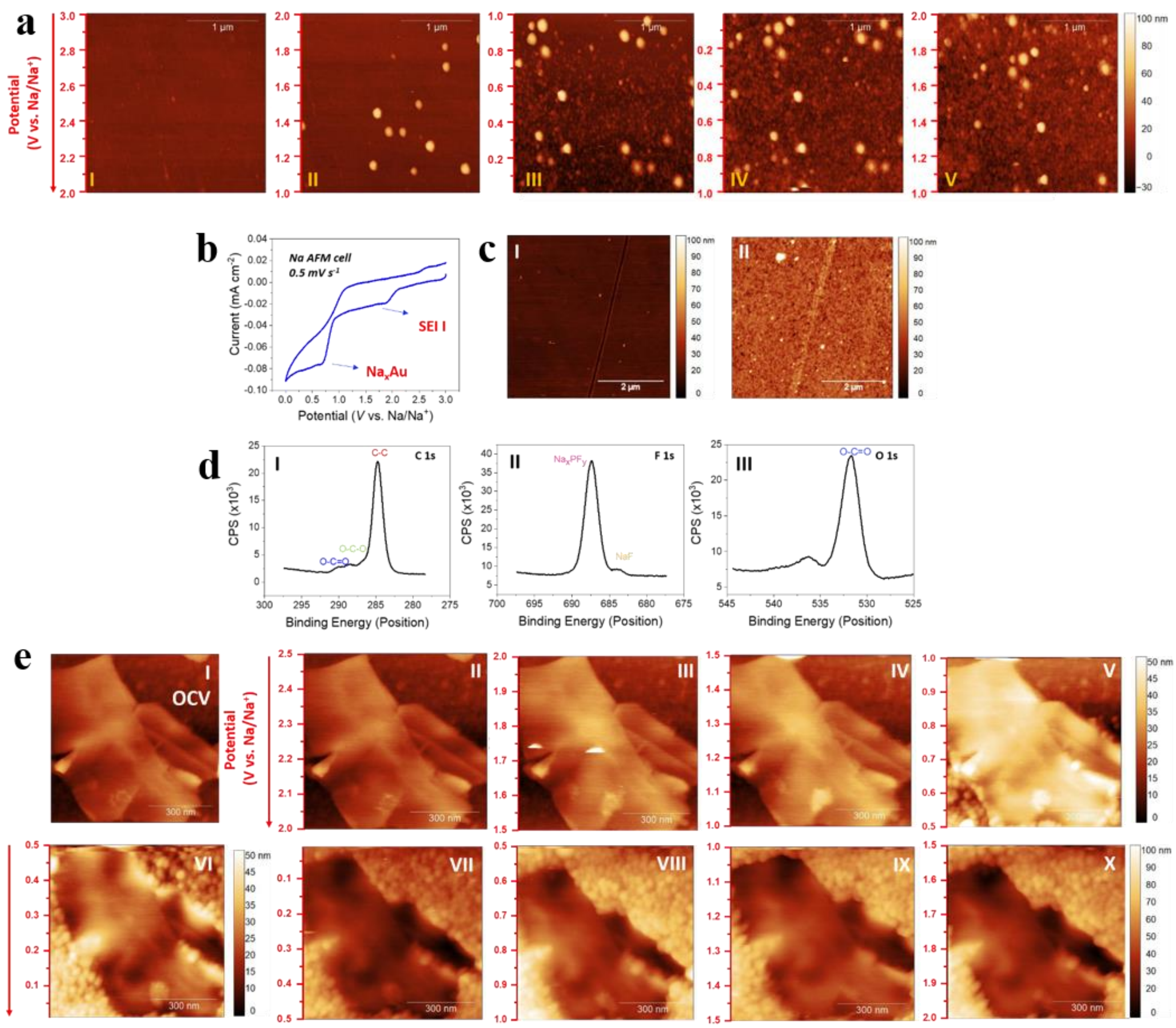

Figure S11: (a) (I ─ V)) *Operando* EC-AFM images of the Au substrate in 2.25 x 2.25 μm scanning in the potential ranges 3 ─ 0.01 V in 1 M $NaPF_6$ EC/DEC electrolyte at 0.5 mV s^{-1}. The scale bar plotted to the right of the image series and all voltages plotted vs. Na/Na^+. (b) The corresponding CV curve. (c) *In situ* EC-AFM images of the Au surface of 5 x 5 $μm^2$ area before (I), and after one sodiation cycle (II). (d) High resolution XPS spectra of the C 1s (I), O 1s (II) and F 1s (III) peaks taken from the surface of a cycled Au substrate. (e) *Operando* EC-AFM

images (700 x 700 nm) of the Au substrate scanning in the potential ranges 2.6 ─ 0.01 V in the same electrolyte and scan rate.

To demonstrate that the formation of an SEI layer, Au-Na alloy and Na plating, on the underlying material has minimal effect on the final morphology of the exfoliated BP, *operando* EC-AFM images during the first cycle of the bare Au/quartz electrode in the same electrolyte were captured and presented in Figure S11a. As the substrate was cycled between 2 – 1 V, large particles begin to deposit on the surface, consistent with the electrical response in the CV Figure S11b, from the formation of an SEI layer. However, at 0.7 V, additional smaller particles nucleate and spread across the surface also consistent with the electrochemical response, which result from the formation of an Au_xNa alloy.[8,9] Finally, although this does not result in large surface changes in the EC-AFM images, close to 0.01 V Na plating has been reported to also occur. [9] The resultant cloudy layer formed maintains its morphology after the anodic sweep. The formation of an SEI layer confirmed by *ex situ* XPS (Figure S11d). However, SEI formation requires direct contact with electrolyte, which would otherwise be prevented with the adhesion of BP to the substrate. This effect shown in Figure S11e, where the morphology of the BP remains intact, despite a distinctive change in the Au substrate. Additionally, the characteristic wrinkling of BP repeatedly seen in Figure 3, Figure 4, Figure S9, and Figure S14 is significantly different to the SEI/Au-Na alloying morphology and consequently not a product of underlying substrate deformation.

In situ **EC-AFM imaging of interfacial processes on Au/Glass electrodes.**

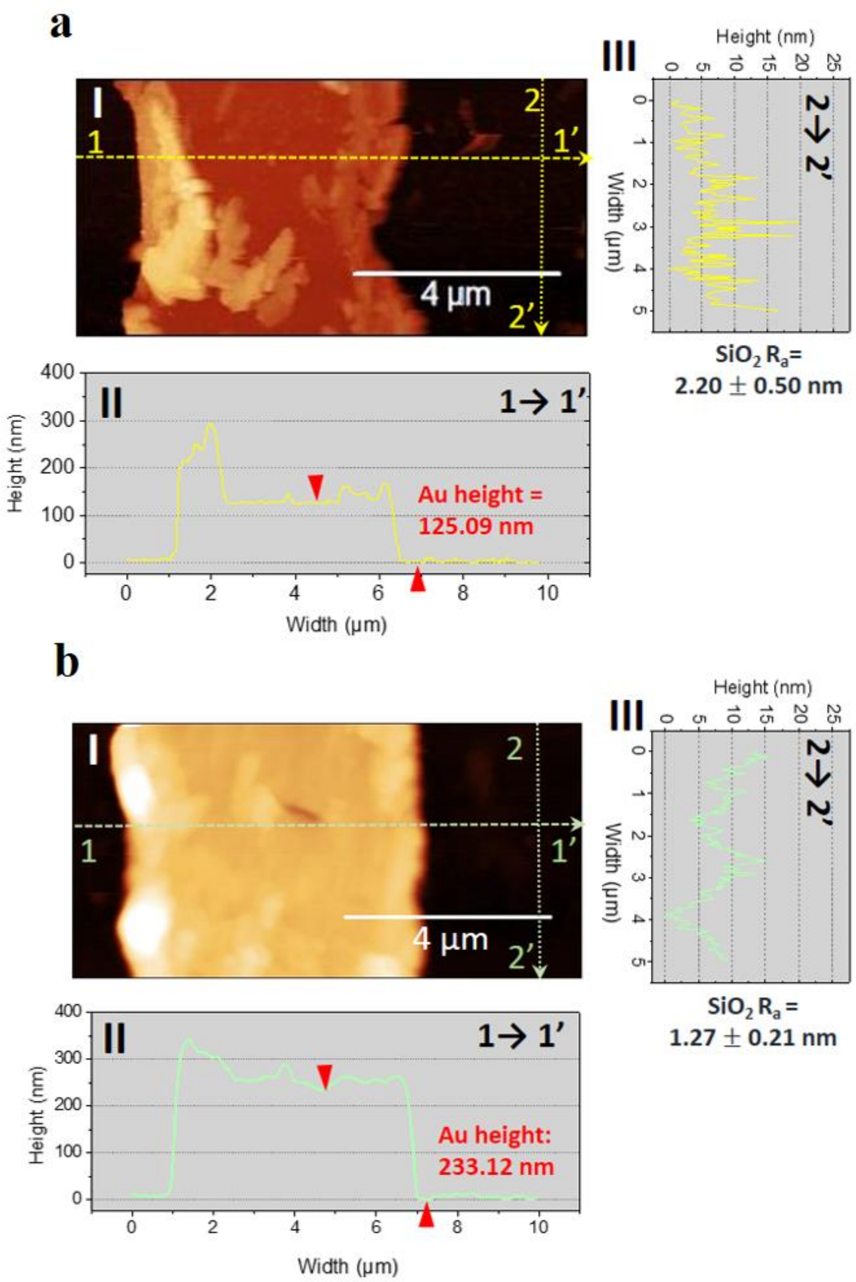

Figure S12. *In situ* EC-AFM images of the BP/Au/glass electrode in 1M $NaPF_6$ EC/DEC electrolyte at (a) OCV and (b) after one cycle (2.5 – 0.01 V vs. Na/Na^+). (b & c II) the plotted height profile taken across the electrode shown with the dotted green arrow 1 →1' and the calculated glass roughness is measured and written below. (b & c III) plots the height profile across the glass only, shown with the green arrow 2 →2'.

***Operando* EC-AFM imaging of interfacial processes on Au, Ag, Cu and Si 100.**

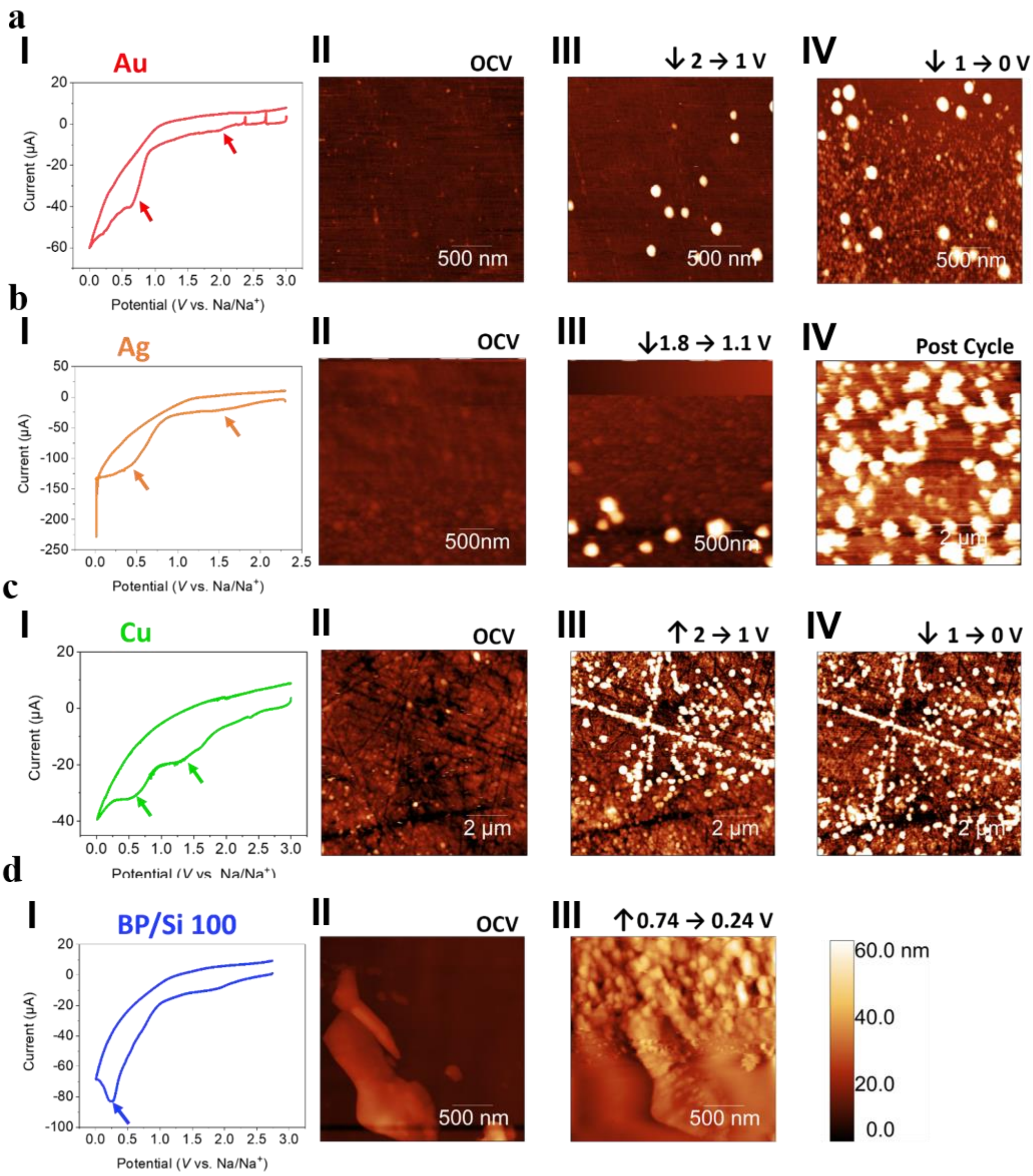

Figure S13: The electrochemical sodiation of Au (a) Ag (b) Cu (c) and BP/Si (d). (a–d (I)) shows the CV of each substrate, (a–d (II–IV)) shows the corresponding *operando* EC-AFM images, captured during the largest surface changes. Black arrows above the images correspond to the direction of the image scan. All potentials quoted vs. Na/Na^+ and all scale bars are shown in the bottom right corner of the images.

Figure S13 shows the electrochemical sodiation of Au (a), Ag (b), Cu (c) and Si (d). Figure S13a−d (I) shows the CV of each substrate, and Figure 13a−d (II−IV) shows the *operando* EC-AFM images, captured during the largest surface changes. In contrast to that reported in literature,[8] the results presented in Figure S13b and c, indicate that both Ag and Cu electrochemically react with sodium in the 1 M $NaPF_6$ EC/DEC electrolyte composition. For Ag, the CV (presented in Figure S13b (I)), shows a small and broad reduction peak occurs between ~ 2.0 – 1.0 V, which corresponds to the initial particle deposition seen in the *operando* EC-AFM Figure S13b (III). This is followed by another peak between ~ 0.75 – 0.01 V, corresponding to the larger surface changes seen in the *in situ* EC-AFM Figure S13b (IV) taken post cycle. Similarly, for Cu, the CV curve demonstrates 2 reduction peaks; at ~ 1.8 – 1.15 V, and ~ 1.0 – 0.2 V, corresponding to the *operando* EC-AFM Figures S13c (III) and c (IV) respectively. Here, a large number of particles develop below 1.8 V, which continue to grow and cover the surface as cycled down to 0.0 V.

The electrochemical sodiation of BP was attempted on crystalline Si 100 (Figure S13d), as this substrate has been shown to be electrochemically inert to towards sodiation.[10] From the CV curve in Figure S13d (I), it was found in fact that a major reduction peak occurs at ~ 0.45 V, resulting in the drastic Si 100 surface changes seen in the *operando* EC-AFM Figure S13c (III). As these changes occur in the precise range of BP alloying, Si 100 surface changes will likely disrupt the BP sodiation imaging, and therefore is not an appropriate substrate. We have demonstrated that out of those tested, no substrate is completely inert to electrochemical sodiation, and that in fact, Au (Figure S13a) presents the least surface changes. Low-level surface roughening below ~0.7 V, which can be seen to be uniform, and the development of a small number of nano-particulate structures are the only changes observed. This demonstrates that it the most suitable substrate. Furthermore, Au has been consistently used as a current collector for *in situ* TEM reports of BP sodiation and lithiation throughout literature. [11−13]

***In situ* EC-AFM imaging of interfacial processes on BP/Ag.**

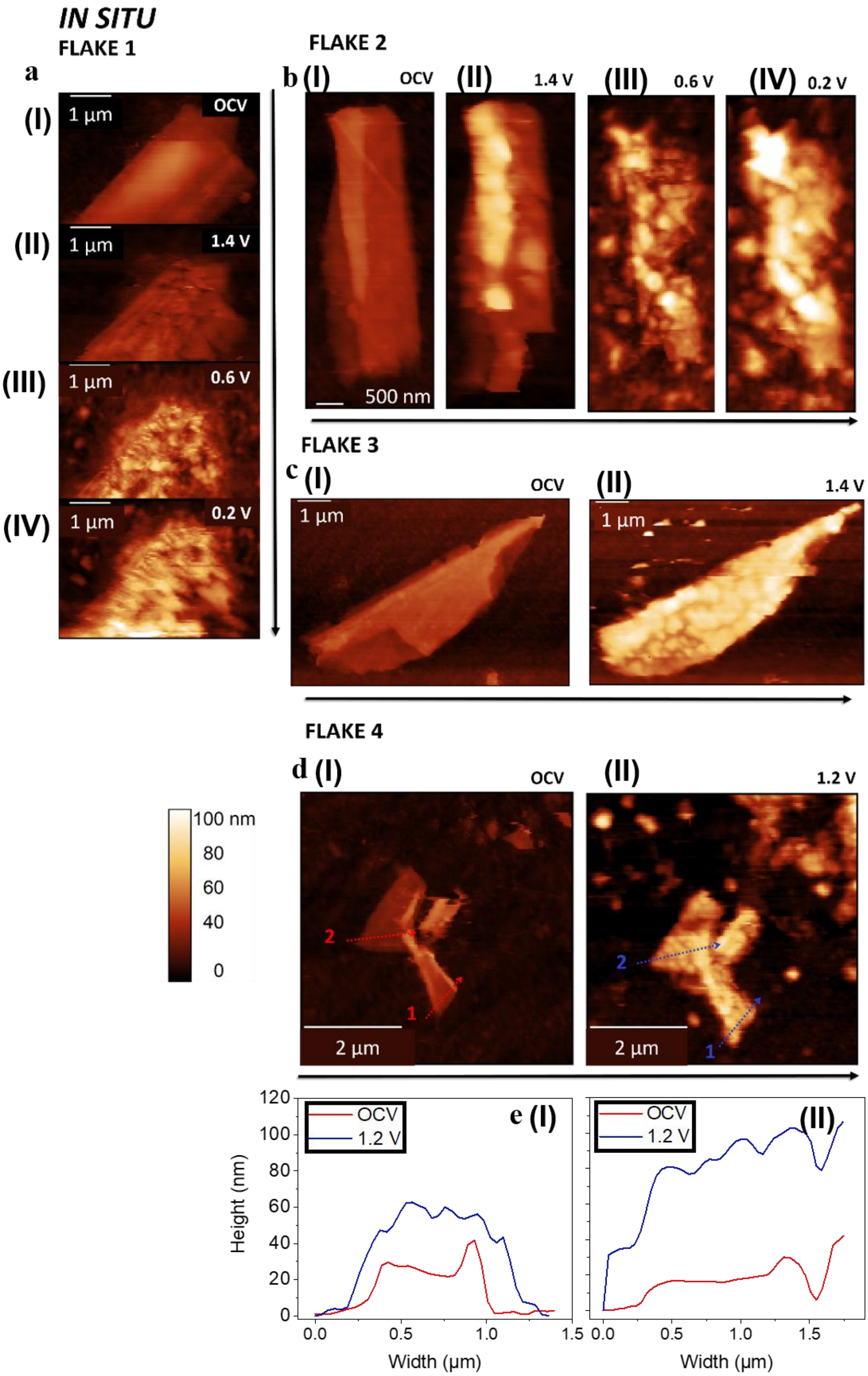

Figure S14: *In situ* EC-AFM images of the BP/Ag electrode in 1M $NaPF_6$ EC/DEC electrolyte. (a−d (I)) is taken at OCV, (a−c (II)) after the cathodic sweep from OVC − 1.4 V, (d (II)) after OCV − 1.2 V, (a−c (III)) is taken at 0.6 V, and (a−c (IV)) at 0.2 V. (All potentials quoted vs. Na/Na^+). (e (I) and (II)) represent the plotted height profile taken across d (I and II), OCV are shown in red, whilst those from the charged BP are in blue shown with the arrow. The red arrows correspond to the region 1 →, and the blue arrow follows 2 →. All scale bars are presented in the corner of each image.

Figure S14 shows the *in situ* EC-AFM images of the BP/Ag sample during the sodiation. Figure S14a−d (I) show the four flakes in electrolyte at OCV, followed by image of each after a cathodic sweep step from OCV to 1.4 V (Figure S4 a−c (II)) and OCV to 1.2V (Figure S14d (II)). From Figure S14a−c (II), it can be seen that the cathodic sweep from OCV to 1.4 V resulted in the formation of an inhomogeneous SEI layer across the BP surface. In particular, Figure S14a and b (II) shows an accumulation of a thin SEI layer or particles at BP basal planes, whilst figure S14c (II) shows the deposition of larger agglomerates of SEI, consistent with our previous EC-AFM Figures 3, 4 and Figure S7. As the potential is lowered to 1.2 V, below the initial sodium intercalation in Figures S14d (II), the BP flake develops nano-wrinkles, consistent with the previous results in Figure 3 and 4 and literature reports.[6,13,16,15,20,22,] These nano-wrinkle are further highlighted through line scan analysis of the height profile taken across 2 regions (Figures S14e (I) and (II)). The height profiles across regions 1 and 2 at OCV are shown in red, whilst those from the charged BP are in blue. An average height increase of ~ 20 nm is observed for region 1 (Figure S14e(I)), and ~65 nm is observed for region 2 (Figure S14e(II)), from the expansion of BP from intercalation. Additionally, peaks and troughs with an average height of ~ 2 nm develop in both line scans, when compared to the smooth and flat

terrace/edges observed at OCV, from the mechanical stress associated with the intercalation mechanism, as is seen consistently in our EC-AFM data.

Further sodiation down to 0.6 V on the Ag substrate results in the development of consistent anisotropic nano-wrinkles of the BP flake, shown in Figure S14a (III), whilst Figure S14b (III) shows an onset disintegration of BP. Nevertheless, both flakes demonstrate a large volume expansion as the potential is swept to below alloying potential at 0.2 V, in Figure S14a and b (IV).

Despite the differences in substrate behaviour between the Au, Sg and interdigitated Au (i.e. BP on Au/glass) surfaces, the behaviour of the active BP during sodiation is consistent, while accepting small particle size/shape/crystal orientation variations, demonstrating that the phenomena observed, including SEI behaviour, intercalation induced stresses and wrinkling and material deterioration upon alloying, are due to changes in the layered phosphorous structure.

***Operando* EC-AFM imaging of SEI formation mechanism on exfoliated BP.**

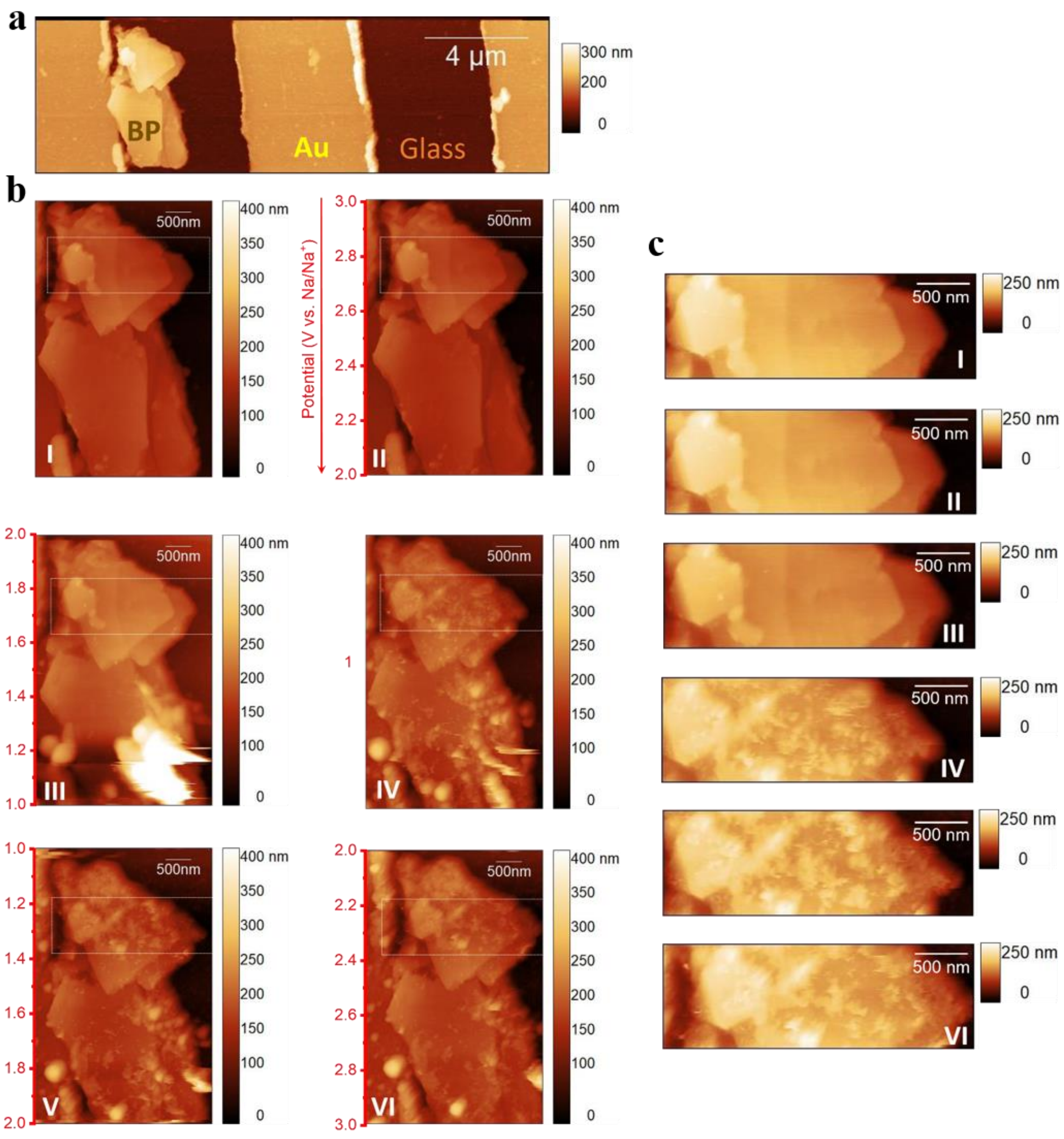

Figure S15: (a) AFM image of an *ex situ* 20 × 6 μm area scan of BP flakes on an Au/Glass current collector substrate. (b (I)) *Operando* EC–AFM images across 10 × 4 μm area on BP/Au/Glass surface in 1 M $NaPF_6$ EC/DEC electrolyte at OVC, and (II — III) captured continuously from during the cathodic scan (3 — 1 V) at 0.5 mV s^{-1}. Each image captures 1 V and scale bars are shown at the right side of the images. (IV) The same flake is captured as the voltage was held at 1 V, and in (IV — VI) during the reverse anodic scan (1 — 3 V). All

corresponding voltages are quoted vs Na/Na^+. (c) Enlarged images of the flake edges taken from the dotted white lined box in (b), with the white line representing 500 nm.

Raman analysis of the cycled BP electrodes.

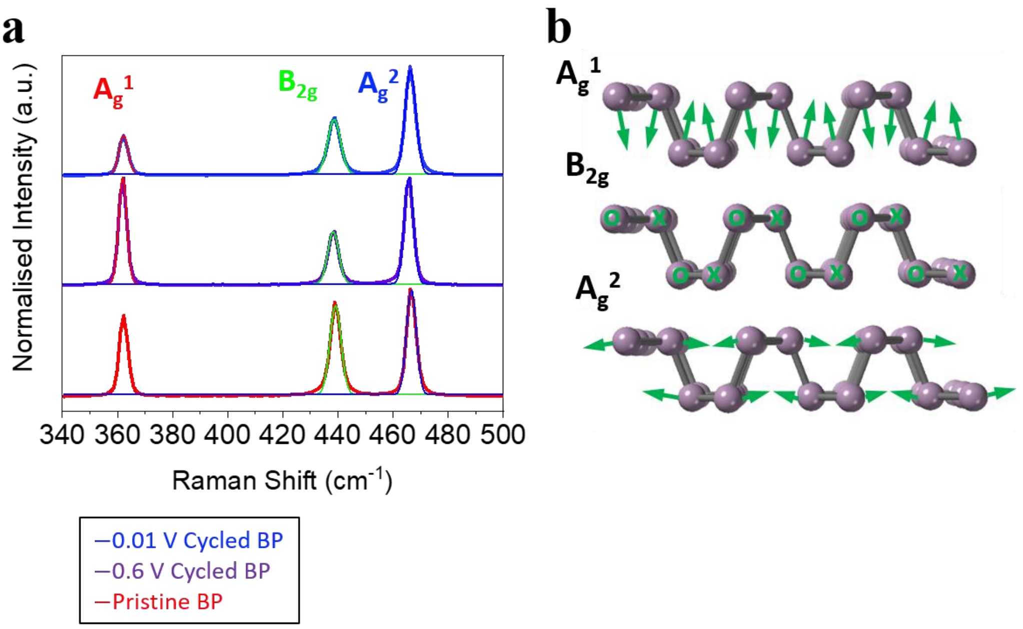

Figure S16: Fitted Raman spectrum of the BP Pristine (red), BP cycled to 0.6 V (Purple) and th marked by cross-hairs in figure inset. Three Lorentzian components are fitted in red, green and blue.

Table S1. Peak positions and full width half maximum (FWHM) values calculated from the fit in Figure S16. Note the instrument resolution has a FWHM of approximately 1.5 cm^{-1}.

	**A^1_g**		**B_{2g}**		**$A_g{}^2$**	
	**Peak position (cm^{-1})**	**FWHM (cm^{-1})**	**Peak position (cm^{-1})**	**FWHM (cm^{-1})**	**Peak position (cm^{-1})**	**FWHM (cm^{-1})**
Pristine BP/Au	362.2 ± 0.04	3.1 ± 0.07	439.1 ± 0.03	4.1 ± 0.07	466.6 ± 0.06	3.5 ± 0.06
0.6 V cycled BP/Au	361.5 ± 0.03	2.9 ± 0.04	437.6 ± 0.05	3.9 ± 0.10	465.0 ± 0.02	3.3 ± 0.10
0.01 V cycled BP/Au	361.0 ± 0.06	3.9 ± 0.15	438.1 ± 0.11	5.6 ± 0.13	465.7 ± 0.06	4.7 ± 0.06

FWHM (Full-width half maximum)

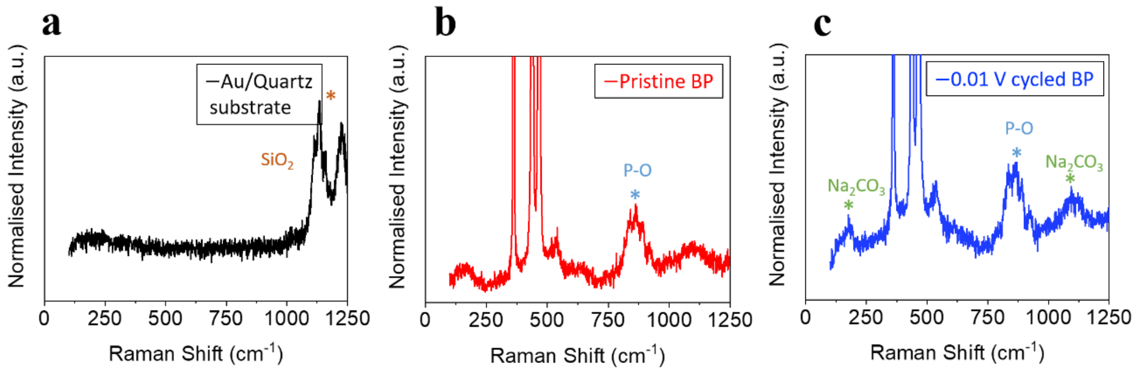

Figure S17: Comparison of the representative Raman spectral intensities acquired for the pristine substrate (a), a substrate deposited with pristine exfoliated BP (b), and the BP deposits cycled in the range 3 – 0.01 V (vs. Na/Na$^+$) (c). The Raman is taken for the cycled BP electrode in the discharged state. All Raman shifts are shown across the range 0 – 1250 cm^{-1}.

Table S2: Raman frequencies measured for Na_2CO_3 on the surface of the 0.6 V cycled BP/Au electrode**.**

	**Na_2CO_3 Raman Assignment**[17,18]							
	T (Na, CO_3)		$(CO_3)^{2-}$ v_4 Doubly degenerate asymmetric (in-plane) bend		$(CO_3)^{2-}$ v_1 Nondegenerate symmetric stretch		v_3 Asymmetric stretching	
	**This study**	**Ref [**Beny (1988)][19]	**This study**	**Ref [**Burgio andClark (2001)][20]	**This study**	**Ref** [Bugzar (2009)][18]	**This study**	**Ref [**Beny (1988)][19]
0.6 V cycled BP/Au	182, 194, 228	111, 131, 149, 171, 189	706	702	~1080	1069-1081	NI*	1421 & 1431

NI* Raman Frequency that appears in literature, but is not detected in our study.

Characterizing the interface of cycled BP electrodes with *Ex situ* XPS.

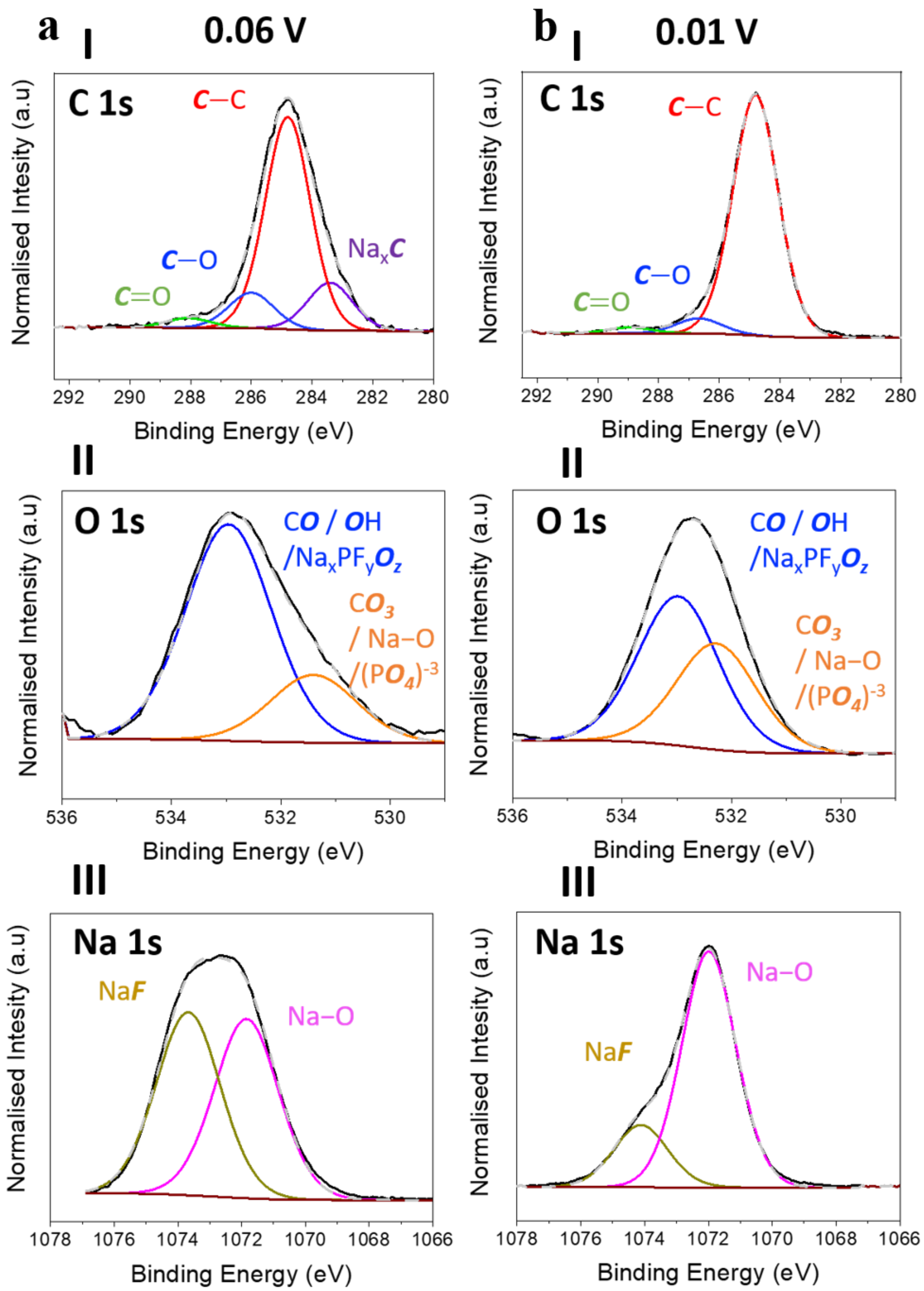

Figure S18: (a) High resolution XPS spectra for for a BP electrode cycled down to 0.6 V, and (b) for a BP electrode cycled down to 0.01 V. For each electrode, the C 1s (I), O 1s (II) and Na 1s (III) regions are plotted.

The C 1s spectra for the cycled electrodes shows peaks at 284.8 eV (C–C, C–H), 286.0 eV (C–O), and 288.3 eV (CO_3) which are typical of carbonate like species that could be assigned to organic species, sodium alkoxides (RCH_2ONa) and Na_2CO_3, generated by the reduction of solvents in SEI. [21,22] An addtitional extremely low binding energy of 283.7 eV is found for electrode cycled to 0.6 V showed was ascribed to C in the sodiated Na_x-C electrode. [23,24] The O 1s spectra presented in Figure S18 a & b (II), are more complicated to deconvolute. The broader peak at 531.3 eV is likely a product of the carbonate like organic components from Na_2CO_3, inorganic compounds containing Na–O that were deprived of PF_6^- solvent complex reduction products such as Na_xPF_y and/or $Na_xPF_yO_z$, and accompanied by phosphate anions at 532.0 eV.[25–28] The peak at 533.2 eV could be related to several overlapping other organic species such as $ROCO_2Na$, CH_3ONa, $(CH_2OCO_2Na)_2$ etc that are known components of NIB SEIs from the double-electron reduction process of EC.[29–31] The Na 1s XPS peak at 1071 eV and 1074 eV further confirmed the existence of inorganic compounds with Na–O Na–F bonds. [32]

Table S3. Atomic percentages of the elemental composition of BP/Au electrode surfaces as a function of cutoff voltage.

**Atomic (%)**	**Pristine BP**	**0.6 V cycled BP/Au**	**0.01 V cycled BP/Au**
C 1s	27	10	22
O 1S	10	10	29
Na 1s	-	17	14
F 1s	-	53	30
P 2p	62	10	5

Elemental compositions are calculated from fitting XPS peak intensities to show relative changes in concentration of species of the BP electrode pristine vs. after cycling to potentials above (0.6 V) and below (0.01 V) alloying, and are summarised in **Table S3**. Changes in the composition of the electrode surface films with cycling are consistent with the deposition of electrolyte products on the anode surface. It can be seen that upon cycling the surface concentration of P decreases while the C/O increase due to the deposition of electrolyte decomposition products. Similarly after cycling concentrations of Na and F are apparent, likely due to contributions from inorganic SEI components.

REFERENCES

(1) Violano, G.; Demelio, G. P.; Afferrante, L. On the DMT Adhesion Theory: From the First Studies to the Modern Applications in Rough Contacts. *Procedia Struct. Integr.* **2018**, *12*, 58–70. https://doi.org/10.1016/j.prostr.2018.11.106.

(2) Derjaguin, B. V.; Muller, V. M.; Toporov, Y. P. Effect of Contact Deformations on the Adhesion of Particles. *J. Colloid Interface Sci.* **1975**, *53* (2), 314. https://doi.org/10.1016/0021-9797(75)90018-1.

(3) Kopycinska-Müller, M.; Geiss, R. H.; Hurley, D. C. Contact Mechanics and Tip Shape in AFM-Based Nanomechanical Measurements. *Ultramicroscopy* **2006**, *106*, 466–474. https://doi.org/10.1016/j.ultramic.2005.12.006.

(4) Watts, M. C.; Picco, L.; Russell-Pavier, F. S.; Cullen, P. L.; Miller, T. S.; Bartuś, S. P.; Payton, O. D.; Skipper, N. T.; Tileli, V.; Howard, C. A. Production of Phosphorene Nanoribbons. *Nature* **2019**, *586*, 216–220. https://doi.org/10.1038/s41586-019-1074-x.

(5) Cresce, A. V.; Russell, S. M.; Baker, D. R.; Gaskell, K. J.; Xu, K. In Situ and Quantitative Characterization of Solid Electrolyte Interphases. *Nano Lett.* **2014**, *14*, 1405–1412. https://doi.org/10.1021/nl404471v.

(6) Baolin Wu, Chunguang Chen, Dmitri L. Danilov, Ming Jiang, Luc H. J. Raijmakers, Rüdiger-A. Eichel, and P. H. L. Influence of the SEI Formation on the Stability and Lithium Diffusion in Si Electrodes. *ACS Omega* **2022**, *7* (36), 32740–32748.

(7) Legrain, F.; Sottmann, J.; Kotsis, K.; Gorantla, S.; Sartori, S.; Manzhos, S. Amorphous (Glassy) Carbon, a Promising Material for Sodium Ion Battery Anodes: A Combined First-Principles and Experimental Study. *J. Phys. Chem. C* **2015**, *119* (24), 13496–13501. https://doi.org/10.1021/acs.jpcc.5b03407.

(8) Sommer, A. Alloys of Gold and Alkali Metals. *Nature* **1943**, *152* (215), 1943.

(9) Tang, S.; Qiu, Z.; Wang, X. Y.; Gu, Y.; Zhang, X. G.; Wang, W. W.; Yan, J. W.;

Zheng, M. Sen; Dong, Q. F.; Mao, B. W. A Room-Temperature Sodium Metal Anode Enabled by a Sodiophilic Layer. *Nano Energy* **2018**, *48*, 101–106. https://doi.org/10.1016/j.nanoen.2018.03.039.

(10) Lim, C. H.; Huang, T. Y.; Shao, P. S.; Chien, J. H.; Weng, Y. T.; Huang, H. F.; Hwang, B. J.; Wu, N. L. Experimental Study on Sodiation of Amorphous Silicon for Use as Sodium-Ion Battery Anode. *Electrochim. Acta* **2016**, *211*, 65–272. https://doi.org/10.1016/j.electacta.2016.06.031.

(11) Cheng, Y.; Zhu, Y.; Han, Y.; Liu, Z.; Yang, B.; Nie, A.; Huang, W.; Shahbazian-Yassar, R.; Mashayek, F. Sodium-Induced Reordering of Atomic Stacks in Black Phosphorus. *Chem. Mater.* **2017**, *29* (3), 1350–1356. https://doi.org/10.1021/acs.chemmater.6b05052.

(12) Sun, J.; Lee, H. W.; Pasta, M.; Yuan, H.; Zheng, G.; Sun, Y.; Li, Y.; Cui, Y. A Phosphorene-Graphene Hybrid Material as a High-Capacity Anode for Sodium-Ion Batteries. *Nat. Nanotechnol.* **2015**, *10*, 980–985. https://doi.org/10.1038/nnano.2015.194.

(13) Xia, W.; Zhang, Q.; Xu, F.; Ma, H.; Chen, J.; Qasim, K.; Ge, B.; Zhu, C.; Sun, L. Visualizing the Electrochemical Lithiation/Delithiation Behaviors of Black Phosphorus by in Situ Transmission Electron Microscopy. *J. Phys. Chem. C* **2016**, *120* (11), 5861–5868. https://doi.org/10.1021/acs.jpcc.5b11218.

(14) Nie, A.; Cheng, Y.; Ning, S.; Foroozan, T.; Yasaei, P.; Li, W.; Song, B.; Yuan, Y.; Chen, L.; Salehi-Khojin, A.; Mashayek, F.; Shahbazian-Yassar, R. Selective Ionic Transport Pathways in Phosphorene. *Nano Lett.* **2016**, *26* (4), 2240–2247. https://doi.org/10.1021/acs.nanolett.5b04514.

(15) Williams, C. L.; Chang, C. C.; Do, P.; Nikbin, N.; Caratzoulas, S.; Vlachos, D. G.; Lobo, R. F.; Fan, W.; Dauenhauer, P. J. Cycloaddition of Biomass-Derived Furans for Catalytic Production of Renewable p -Xylene. *ACS Catal.* **2012**, *2* (6), 935–939.

https://doi.org/10.1021/cs300011a.

(16) Hembram, K. P. S. S.; Jung, H.; Yeo, B. C.; Pai, S. J.; Kim, S.; Lee, K. R.; Han, S. S. Unraveling the Atomistic Sodiation Mechanism of Black Phosphorus for Sodium Ion Batteries by First-Principles Calculations. *J. Phys. Chem. C* **2015**, *119* (27), 15041–15046. https://doi.org/10.1021/acs.jpcc.5b05482.

(17) Brooker, M. H.; Bates, J. B. Raman and Infrared Spectral Studies of Anhydrous Li2CO3 and Na 2CO3. *J. Chem. Phys.* **1971**, *54*, 4788. https://doi.org/10.1063/1.1674754.

(18) Buzgar, N.; Apopei, A. I. *The Raman Study of Certain Carbonates*; The Publishing House of the Alexandru Ioan Cuza University: Iași, 2009; Vol. 2.

(19) Beny, C. Base de Données de Spectres Raman Natrite. *Société Française Minéralogie Cristallogr.* **1988**.

(20) Burgio, L.; Clark, R. J. H. Library of FT-Raman Spectra of Pigments, Minerals, Pigment Media and Varnishes, and Supplement to Existing Library of Raman Spectra of Pigments with Visible Excitation. *Spectrochim. Acta - Part A Mol. Biomol. Spectrosc.* **2001**, *57* (7), 1491–1521. https://doi.org/10.1016/S1386-1425(00)00495-9.

(21) Dedryvère, R.; Martinez, H.; Leroy, S.; Lemordant, D.; Bonhomme, F.; Biensan, P.; Gonbeau, D. Surface Film Formation on Electrodes in a LiCoO2/Graphite Cell: A Step by Step XPS Study. *J. Power Sources* **2007**, *174* (2), 462–468. https://doi.org/10.1016/j.jpowsour.2007.06.033.

(22) Bai, P.; Han, X.; He, Y.; Xiong, P.; Zhao, Y.; Sun, J.; Xu, Y. Solid Electrolyte Interphase Manipulation towards Highly Stable Hard Carbon Anodes for Sodium Ion Batteries. *Energy Storage Mater.* **2020**, *25*, 324–333. https://doi.org/10.1016/j.ensm.2019.10.006.

(23) Eshetu, G. G.; Diemant, T.; Hekmatfar, M.; Grugeon, S.; Behm, R. J.; Laruelle, S.; Armand, M.; Passerini, S. Impact of the Electrolyte Salt Anion on the Solid Electrolyte

Interphase Formation in Sodium Ion Batteries. *Nano Energy* **2019**, *55*, 327–340. https://doi.org/10.1016/j.nanoen.2018.10.040.

(24) Ciosek Högström, K.; Malmgren, S.; Hahlin, M.; Rensmo, H.; Thébault, F.; Johansson, P.; Edström, K. The Influence of PMS-Additive on the Electrode/Electrolyte Interfaces in LiFePO4/Graphite Li-Ion Batteries. *J. Phys. Chem. C* **2013**, *117* (45), 23476–23486. https://doi.org/10.1021/jp4045385.

(25) van der Heide, P. *X-Ray Photoelectron Spectroscopy: An Introduction to Principles and Practices*, 264th ed.; John Wiley & Sons: Hoboken, 2011. https://doi.org/10.1002/9781118162897.

(26) Wang, Z.; Yang, H.; Liu, Y.; Bai, Y.; Chen, G.; Li, Y.; Wang, X.; Xu, H.; Wu, C.; Lu, J. Analysis of the Stable Interphase Responsible for the Excellent Electrochemical Performance of Graphite Electrodes in Sodium-Ion Batteries. *Small* **2020**, *16* (51), 2003268. https://doi.org/10.1002/smll.202003268.

(27) Li, K.; Zhang, J.; Lin, D.; Wang, D. W.; Li, B.; Lv, W.; Sun, S.; He, Y. B.; Kang, F.; Yang, Q. H.; Zhou, L.; Zhang, T. Y. Evolution of the Electrochemical Interface in Sodium Ion Batteries with Ether Electrolytes. *Nat. Commun.* **2019**, No. 10, 725. https://doi.org/10.1038/s41467-019-08506-5.

(28) Zhang, H.; Wang, D.; Shen, C. In-Situ EC-AFM and Ex-Situ XPS Characterization to Investigate the Mechanism of SEI Formation in Highly Concentrated Aqueous Electrolyte for Li-Ion Batteries. *Appl. Surf. Sci.* **2020**, *507*, 145059. https://doi.org/10.1016/j.apsusc.2019.145059.

(29) Bai, P.; He, Y.; Xiong, P.; Zhao, X.; Xu, K.; Xu, Y. Long Cycle Life and High Rate Sodium-Ion Chemistry for Hard Carbon Anodes. *Energy Storage Mater.* **2018**, *13*, 274–282. https://doi.org/10.1016/j.ensm.2018.02.002.

(30) Aurbach, D. Review of Selected Electrode-Solution Interactions Which Determine the Performance of Li and Li Ion Batteries. *J. Power Sources* **2000**, *89* (2), 206–218.

https://doi.org/10.1016/S0378-7753(00)00431-6.

(31) Xu, S. D.; Zhuang, Q. C.; Wang, J.; Xu, Y. Q.; Zhu, Y. B. New Insight into Vinylethylene Carbonate as a Film Forming Additive to Ethylene Carbonate-Based Electrolytes for Lithium-Ion Batteries. *Int. J. Electrochem. Sci.* **2013**, *8*, 8058–8076.

(32) Hy, S.; Felix; Chen, Y. H.; Liu, J. Y.; Rick, J.; Hwang, B. J. In Situ Surface Enhanced Raman Spectroscopic Studies of Solid Electrolyte Interphase Formation in Lithium Ion Battery Electrodes. *J. Power Sources* **2014**, *256*, 324–328. https://doi.org/10.1016/j.jpowsour.2014.01.092.
